# Combinatorial therapy with BAR502 and UDCA resets FXR and GPBAR1 signaling and reverses liver histopathology in a model of NASH

**DOI:** 10.1038/s41598-023-28647-4

**Published:** 2023-01-28

**Authors:** Silvia Marchianò, Michele Biagioli, Elva Morretta, Cristina Di Giorgio, Rosalinda Roselli, Martina Bordoni, Rachele Bellini, Ginevra Urbani, Carmen Massa, Maria Chiara Monti, Angela Zampella, Eleonora Distrutti, Stefano Fiorucci

**Affiliations:** 1 Department of Medicine and Surgery, University of Perugia, Perugia, Italy; 2 Department of Pharmacy, University of Salerno, Salerno, Italy; 3 Department of Pharmacy, University of Naples Federico II, Naples, Italy; 4 Azienda Ospedaliera di Perugia, Perugia, Italy; 5 Department of Medicine and Surgery, University of Perugia, Perugia, Italy

**Keywords:** Diseases, Gastroenterology

## Abstract

Non-alcoholic steatosis (NAFLD) and steatohepatitis (NASH) are two highly prevalent human disorders for which therapy remains suboptimal. Bile acids are signaling molecules acting on two main receptors the Farnesoid-x-receptor (FXR) and G protein coupled receptor GPB AR1. Clinical trials have shown that FXR agonism might result in side effects along with lack of efficacy in restoring liver histopathology. For these reasons a multi-targets therapy combined FXR agonists with agent targeting additional molecular mechanisms might have improved efficacy over selective FXR agonists. In the present study we have compared the effects of BAR502, a dual FXR/GPBAR1 ligand) alone or in combination with ursodeoxycholic acid (UDCA) in a model of NAFLD/NASH induced by feeding mice with a Western diet for 10 weeks. The results demonstrated that while BAR502 and UDCA partially protected against liver damage caused by Western diet, the combination of the two, reversed the pro-atherogenic lipid profile and completely reversed the histopathology damage, attenuating liver steatosis, ballooning, inflammation and fibrosis. Additionally, while both agents increased insulin sensitivity and bile acid signaling, the combination of the two, modulated up top 85 genes in comparison of mice feed a Western diet, strongly reducing expression of inflammatory markers such as chemokines and cytokines. Additionally, the combination of the two agents redirected the bile acid metabolism toward bile acid species that are GPBAR1 agonist while reduced liver bile acid content and increased fecal excretion. Together, these data, highlight the potential role for a combinatorial therapy based on BAR502 and UDCA in treating of NAFLD.

## Introduction

Non-alcoholic fatty liver disease (NAFLD), is a fast growing clinical problems worldwide, due to excessive hepatic lipid accumulation that is not related to alcohol intake^1,2^. NAFLD encompasses a spectrum of conditions, ranging from simple steatosis, a relatively benign condition, to NASH, which can progress to liver fibrosis, cirrhosis or hepatocellular carcinoma^3–5^. NASH is a multisystem disease, affecting several extra-hepatic organs and regulatory pathways, and is already the most common causes of chronic liver disease in adults and obese children in several countries^6,7^. Despite NAFLD/NASH is attracting a global research interest, therapy remains suboptimal^8,9^.

In the past decade, it has been demonstrated that bile acid signaling has important physio-pathologic and therapeutic implications in NAFLD/NASH. Bile acids are a large family of molecules with a steroidal structure, synthesized in liver from cholesterol, exerting a non-dispensable role in the absorption of dietary lipids, cholesterol and fat-soluble vitamins^10,11^. Beside this function, however, bile acids are signaling molecules exerting an essential role in regulating their own synthesis, uptake and secretion as well as control of the synthesis and excretion of cholesterol, lipid and glucose metabolism^9,12^. In animals, such as rodents, fecal excretion of bile acids represents a major pathway for cholesterol disposal. Despite this pathway is significantly less developed in humans, animal models are widely used to profile metabolic pathways as well as therapeutic approaches to NAFLD and NASH^13^. Conversion of cholesterol into bile acids takes place in the liver, and is tightly regulated, both in humans and mice, by various transcription factors, such as the Farnesoid-x-receptor (FXR), a master gene that directly or indirectly modulates the expression of the two rate limiting enzymes involved in bile acid synthesis in the liver, the cyp7a1 and cyp8b1^14^. These two genes, govern essential steps in the conversion of cholesterol, in the two primary bile acids, chenodeoxycholic acid (CDCA) and cholic acid (CA). Both the two primary bile acids in humans, act as FXR agonists^12^. Thus activation of FXR in hepatocytes represses the activity of CYP7A1 and CYP8B1^15^, reducing the synthesis of endogenous bile acids, a mechanisms that limit the accumulation of bile acids in the liver, but also hampers the ability of humans to promote disposal of cholesterol through this pathway. In addition, activation of FXR regulates the synthesis of BSEP and promotes the excretion of bile acids from hepatocytes into the bile, while reduces their uptake from the systemic circulation, by inhibiting the expression/synthesis of NTCP^16^. Some of these activities are mediated by the transcription of the small heterodimer partner (SHP), an atypical nuclear receptor that lacks the DNA binding domain and function as a co-repressor for several genes, including CYP7A1 and CYP8B1 and NTCP^16,17^. In addition, FXR activation in the intestinal epithelial cells, promotes the release of the fibroblast growth factor (FGF) 19 (FGF15 is the mouse ortholog)^18^, an ileal peptide hormone that inhibits the bile acid synthesis after binding to a FGF receptor(FGFR)4/βklotho complex in the hepatocytes membrane, promoting the repression of CYP7A1^19^, establishing a tightly regulated mechanism that modulate bile acid synthesis. In contrast to primary bile acids, secondary bile acids (lithocholic- and deoxycholic-acid, LCA and DCA), formed in intestine from the 7α-dehydroxylation of CA and CDCA, preferentially bind and activate a cell membrane receptor, the G-protein bile acid receptor (GPBAR1 also known as TGR5), that is poorly expressed by hepatocytes, but is well represented in muscle cells and adipose tissues^20,21^. Activation of GPABR1^22^ in the abdominal adipose tissues promotes the transition from white adipose tissues (WAT) toward a beige/brown (BAT) phenotype^23^ and increase energy expenditure. In addition, GPBAR1 promotes the release of glucagon like peptide (GLP)-1 from ileal L cells^12,20^. Because the regulatory effects bile acids exert on lipid and glucose homeostasis in various tissues, several steroidal and non-steroidal ligands of FXR/GPBAR1 have been developed for the treatment of NASH^12^. However, while some of the agents have been advanced though clinical studies, several side effects have been observed, the most common of which are pruritus that typically occurs with obeticholic acid^24^ in dose/dependent manner, along with the worsening of lipid lipoprotein profile toward a more proatherogenic lipid profile^25,26^. Obeticholic acid has also been associated to a cluster of hepatic decompensation in cirrhotic patients^27^. In addition to these side effects, all FXR ligands have been only partially effective in reducing liver steatosis and ballooning scores or fibrosis^28–31^, suggesting that combination therapies might be required to improve efficacy and reduce side effects.

BAR502, is a dual FXR and GPBAR1 agonist^32–34^, that is currently advanced to clinical stage, that was shown effective in reducing steatosis and fibrosis in rodents model of NAFLD and NASH. As mentioned above, however, this agent did not completely reverse liver damage and exerts no effects of lipid protein profile in mice feed a high fat, high cholesterol diet. Because in clinical settings an amelioration of lipid profile represents a major therapeutic goal, we have decided to investigate whether a combination of BAR502 withy ursodeoxycholic acid (UDCA), could improve efficacy of the dual FXR/GPBAR1 ligand. UDCA is a CDCA derivative in humans and is a choleretic and anti-inflammatory agent, clinically approved for the treatment of primary biliary cholangitis (PBC)^35^. The effect of UDCA in NAFLD patients, however, is controversial. Thus, while some studies have reported improvements in liver function tests, and steatosis scores^36,37^, others have failed to show improvements compared with the placebo treatment^38,39^. However, UDCA has been used in association to obeticholic acid in PBC patients, and is a highly safe and relative un-expensive agent^40^.

In the present study we have investigated the effects of BAR502 and UDCA alone or in combination in model of NAFLD/NASH induced by feeding mice a HFD and fructose. Our results demonstrated that a combinatorial approach results a robust remodeling of plasma lipid profile along with a complete reversion of liver damage.

## Results

### A combination therapy with BAR502 and UDCA protects against body weight gain, alterations in glucose levels and metabolic changes caused by Western diet

**Figure 1:**
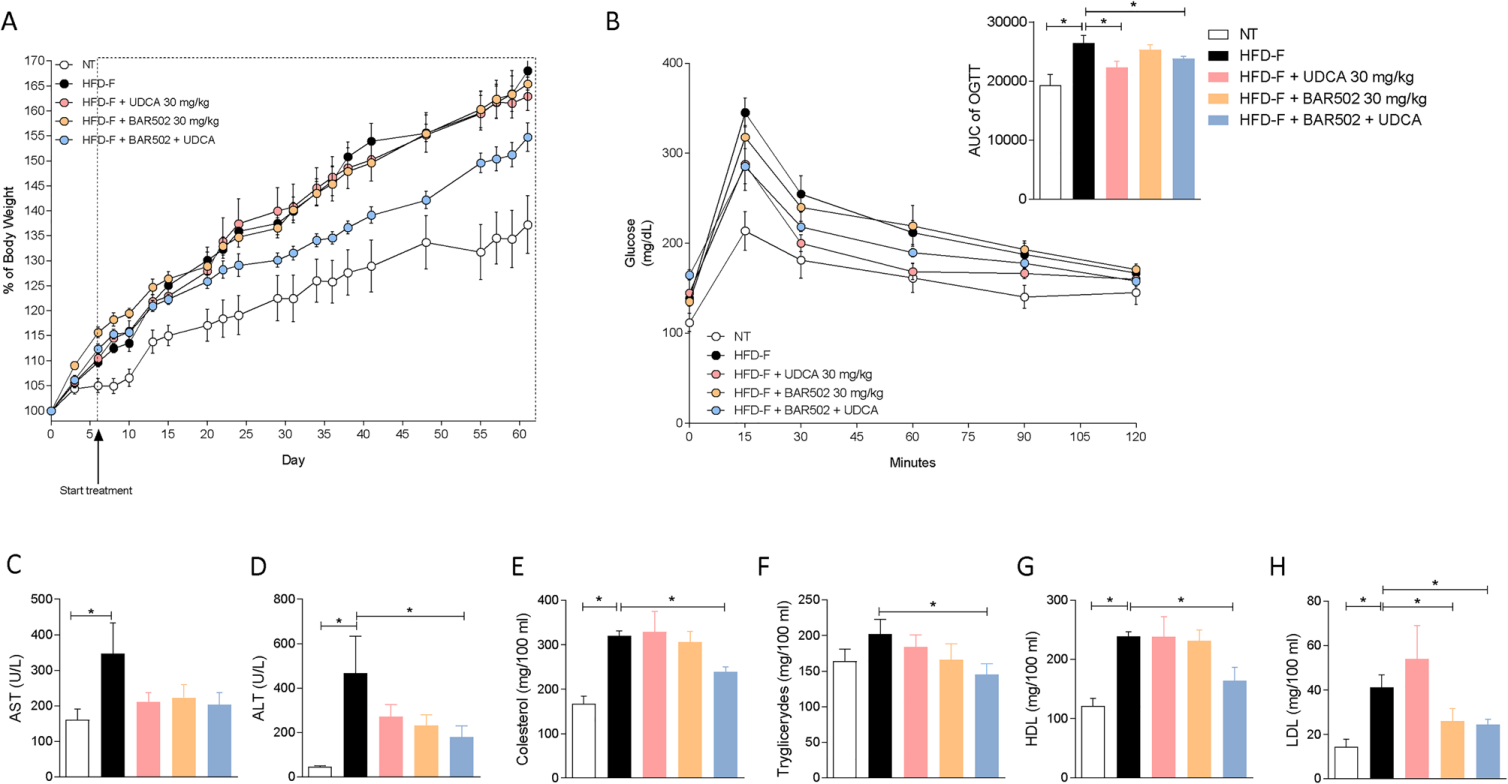
Gpbar1+/+ male mice were fed an HFD-F or normal chow diet for 8 weeks. Mice were randomized to receive the HFD-F alone or the HFD-F in combination with UDCA 30 mg/kg or BAR502 30 mg/kg or BAR502 + UDCA, by gavage for 7 weeks. (**A**) Changes in body weight (g) in mice were assessed weekly. (**B**) Glucose plasma levels in response to oral glucose tolerance test and area under the curve (AUC) values of glucose plasma levels expressed in arbitrary units. (**C**–**H**) Serum levels of AST, ALT, Cholesterol, Triglycerides, HDL and LDL. Biochemistry values were measured at the end of the study. The data are mean ± SE of 7–10 mice (*p < 0.05).

To gain insights on the therapeutic potential of BAR502 and UDCA, we have first assessed whether the two agents were effective in reversing liver steatosis induced by 8-weeks feeding mice with a high caloric diet enriched in cholesterol and fructose in drinking water (the so-called Western diet). All mice were feed this diet for one week and then randomized to receive one of the following treatments, no treatment, BAR502 30 mg/kg, UDCA 30 mg/kg or the combination of the two agents for 8 weeks. All mice have a similar body weight at the beginning of the study, but mice feed a Western diet gained approx. 35% more weight than mice feed a standard chow diet (Fig. 1A).

Body weight gain was significantly attenuated treating mice with the combination of BAR502 and UDCA, while the two agents separately showed no effects.

On the 8th week of the study a glucose tolerance test was carried out to investigate the glucose levels in the various experimental groups and as shown in Fig. 1B, we found that mice feed a Western diet were hyperglycemic at baseline and showed a severe alteration in glucose levels as demonstrated by the value of the area under the curve of OGTT (Fig. 1B, inset) in comparison to mice feed a standard diet. As shown in Fig. 1B, however, treating mice with UDCA alone or in combination with BAR502 reversed this pattern. To further characterize whether feeding a Western diet promotes the development of a NAFLD-like biochemistry profile, we have then measured serum liver enzymes and lipid profiles. As shown in Fig. 1C–H, while feeding a Western diet resulted in hepatocytes injury, as shown by changes in AST and ALT plasma levels, and development of a pro-atherogenic lipid profile, i.e. increased levels of cholesterol, triglycerides and HDL and LDL; this pattern was fully reversed by the combination of UDCA and BAR502, BAR502 also reduced the LDL levels, without reducing HDL, a common side effect observed with selective FXR agonists^41^.

### A combination therapy with BAR502 and UDCA reverses histology features of liver steatosis, ballooning and fibrosis in mice feed a Western diet

**Figure 2:**
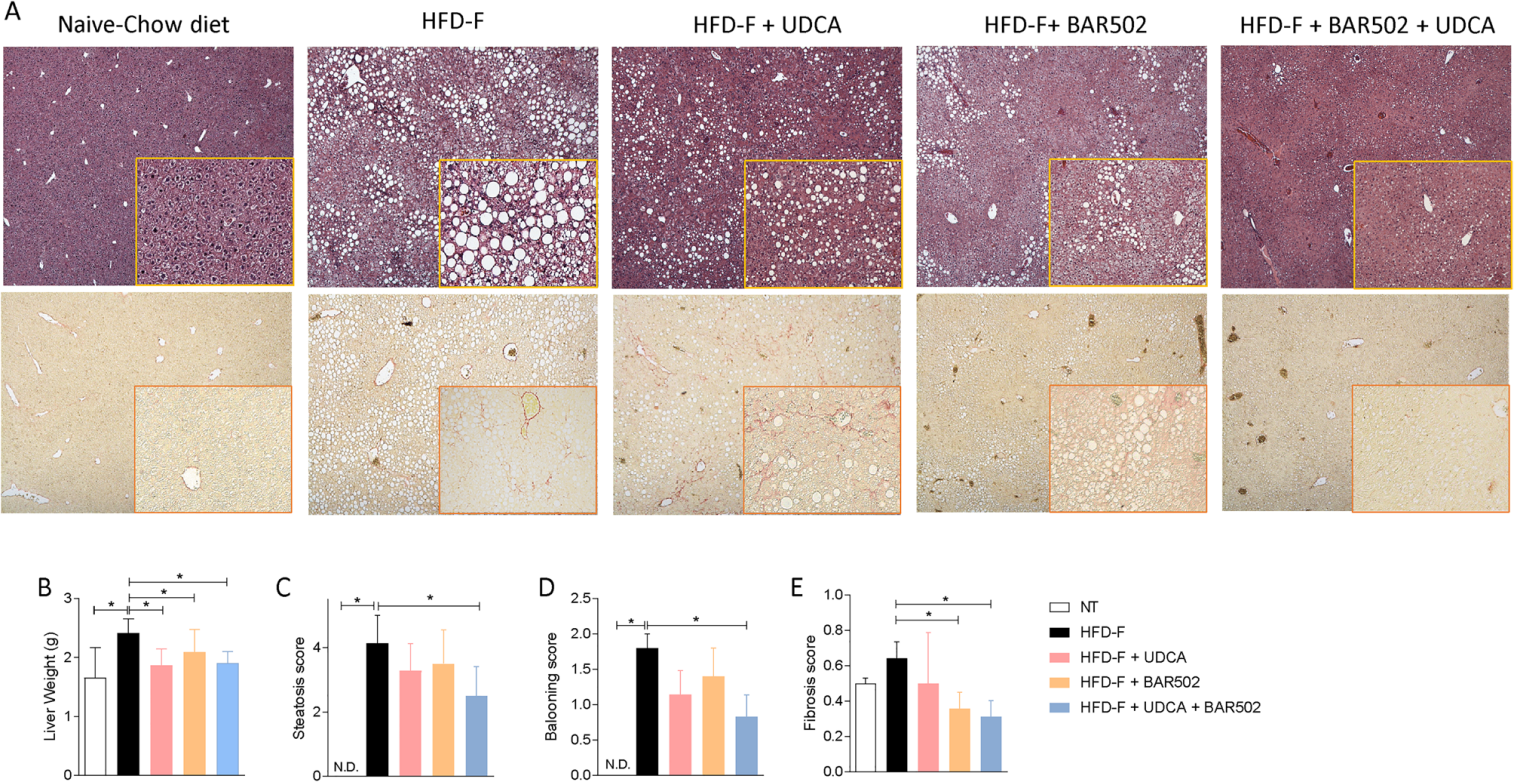
(**A**) H&E staining and Sirius Red staining of liver tissues obtained at the end of the study. Original magnification, × 10. (**B**) Liver weight. NASH severity was scored by assessing, (**C**) the liver steatosis score, (**D**) ballooning and (**E**) fibrosis score in at least 5 different fields per liver in a blinded manner.

We have then investigated the liver histopathology changes caused by feeding mice the Western diet. For these purposes H&E-stained liver sections were assessed at 10 × magnification and severity of liver steatosis scored for steatosis, inflammation and hepatocytes ballooning as described previously^25,33,34^. The results of these histopathology analyses demonstrated that while mice fed a chow diet had a normal liver histology with normal lobular architecture without signs of inflammation or accumulation of lipid droplets in hepatocytes (Fig. 2A,B), major morphological changes were documented in mice fed a Western diet, that developed hepatic steatosis accompanied by severe hepatocyte’s ballooning (Fig. 2C,D).

As shown in Fig. 2C,D, these pathological changes were partially mitigated by UDCA and BAR502, but were almost completely abrogated by the co-treatment with BAR502 and UDCA. Of relevance, the combination therapy effectively attenuated development of hepatocyte’s ballooning and liver fibrosis (Fig. 2E), that was also reversed by BAR502 but not by UDCA alone, as observed in several clinical trials^42^.

**Figure 3:**
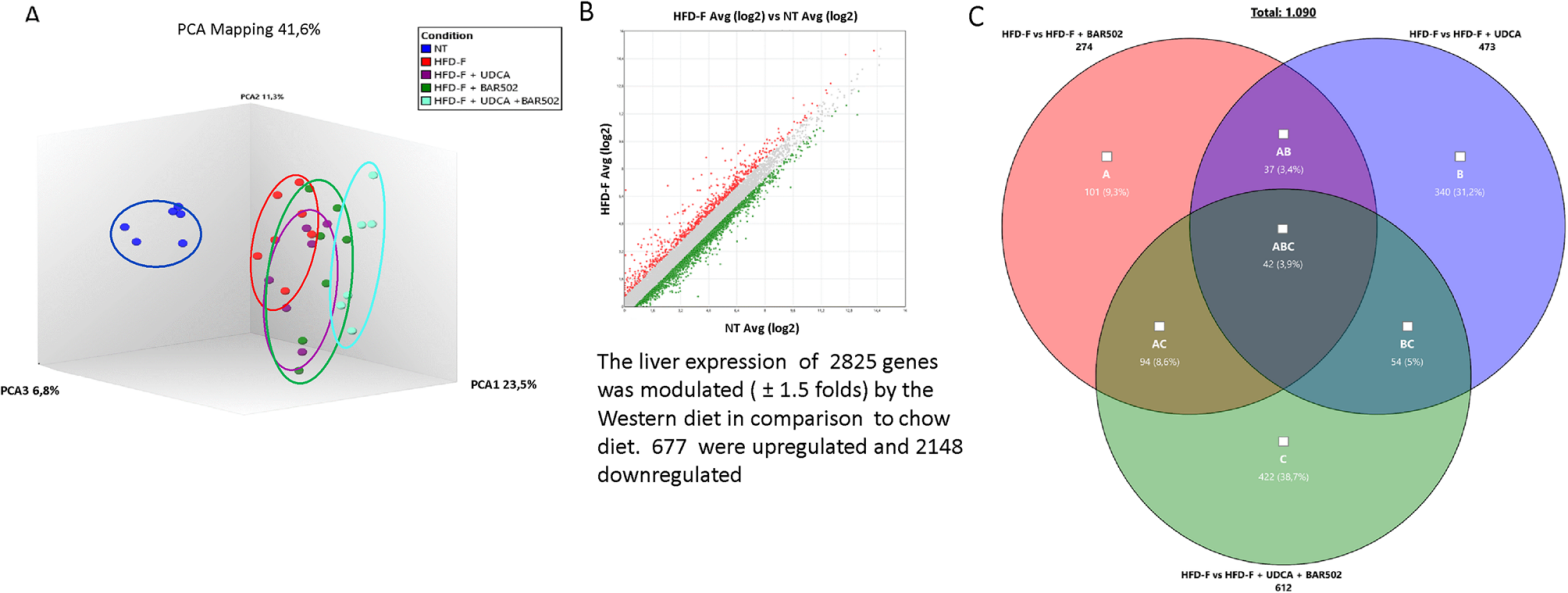
RNA-seq of BAR502, UDCA and BAR502 + UDCA treated mice. BAR502, 30 mg/kg/day, UDCA and their combination were administered by gavage in C57BL/6J male mice starting on day 10 of high fat diet (HFD) and fructose for an additional 7 weeks. (**A**) Quantitative β analysis of PCoA that showed the dissimilarity between the experimental group, (**B**) Scatter plot of genes modulated by HFD-F vs NT experimental group and (**C**) Venn diagram of differentially expressed genes showing the overlapping regions (identified as ABC, AC, AB, and BC sets) between the three experimental groups of mice (Fold Change < − 2 or > 2, p value < 0.05).

To identify molecular mechanisms supporting the beneficial effects exerted by the various treatments, we have carried out a RNAseq analysis of the liver transcriptomes in the five experimental groups (Fig. 3A).

As expected, feeding mice a Western diet resulted in an extensive remodelling of liver transcriptome with up to 2825 genes whose expression was up- or down-regulated (677 and 2148 respectively) in comparison to mice feed a regular chow diet (± 1.5 folds change) (Fig. 3B). This pattern was only partially modulated by the pharmacological treatments. However, while treating mice with BAR502 and UDCA modulated the expression of 274 and 473 genes (± 1.5 fold changes), the combination of the two agents modulated the expression of 612 genes, 422 of which were not modulated neither of BAR502 nor by UDCA alone (Fig. 3C). Using a more stringent protocol, i.e. only genes whose expression was modulated at least two folds, we found that co-treating mice with BAR520 and UDCA modulated the expression of 121 genes, 89 of which were modulated only by the combination of BAR502 and UDCA (Supplementary Table S1). Further analysis of these pathways revealed that only 7 genes were modulated by the three treatments (Table 1), while 23 genes were shared by BAR502-UDCA and BAR502 alone, and only two by BAR502-UDCA and UDCA alone (Supplementary Tables S2 and S3).

**Table 1:** Genes modulated by the three analyzed treatments (downregulated genes in bold, upregulated genes in italics).

ID	HFD-F + UDCA + BAR502 Avg (log2)	HFD-F + UDCA Avg (log2)	HFD-F + BAR502 Avg (log2)	HFD-F Avg (log2)	Fold Change HFD-F + UDCA + BAR502 vs HFD-F	P-val	Fold Change HFD-F + UDCA vs HFD-F	P-val	Fold Change HFD-F + BAR502 vs HFD-F	P-val
Cfd	1.21	3.75	1.15	6.77	− **47.02**	6.83E−07	− **8.08**	0.023	− **49.01**	1.66E−06
Cidea	0.12	1.16	0.13	2.96	− **7.18**	4.81E−07	− **3.49**	0.0001	− **7.12**	9.80E−07
Fgf11	1.61	1.77	1.4	3.61	− **4**	9.38E−07	− **3.59**	1.04E−06	− **4.62**	6.40E−08
Elovl3	8.74	9.01	9.08	10.19	− **2.74**	0.0009	− **2.28**	0.0088	− **2.17**	0.0042
Dbp	7.5	6.88	7.04	5.37	*4.37*	0.001	*2.86*	0.0095	*3.18*	0.0092
Per3	5.19	4.2	4.63	2.95	*4.71*	0.0002	*2.37*	0.0369	*3.2*	0.0143
Usp2	6.14	4.85	4.77	3.63	*5.69*	3.54E−08	*2.33*	0.0002	*2.21*	0.0001

**Figure 4:**
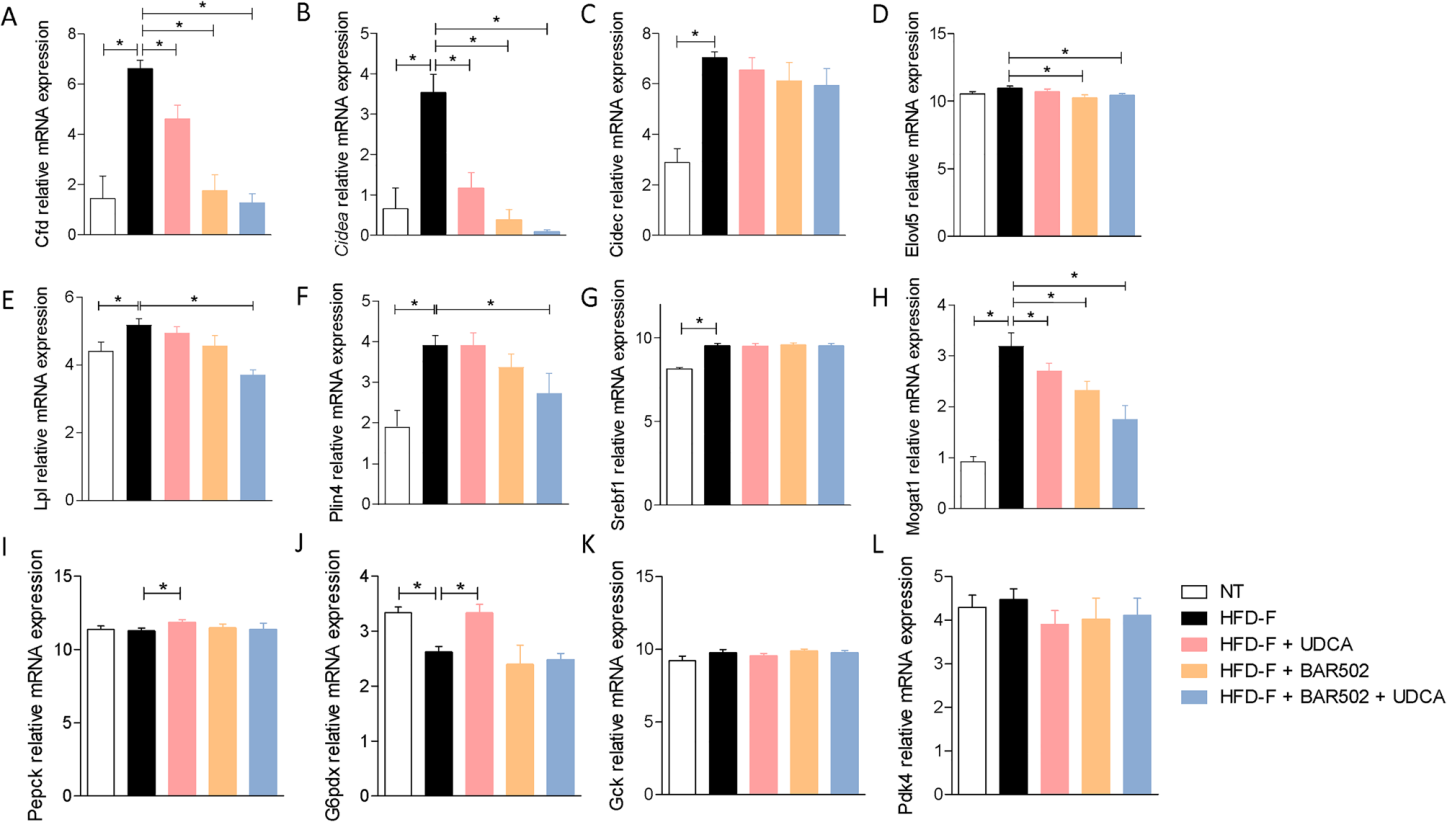
Expression levels of genes involved in lipid metabolism, extracted from RNA-seq analysis: (**A**) Cfd, (**B**) Cidea, (**C**) Cidec, (**D**) Elovl5, (**E**) Lpl, (**F**) Plin4, (**G**) Srebf1, (**H**) Mogat, (**I**) Pepck, (**J**) G6pdx (**K**) Gck, (**L**) Pdk4.

Among genes that were downregulated by the three treatments, Cdf (also known as adipsin) (Fig. 4A) was the most downregulated gene (up to 49 folds by BAR502 alone or in combination with UDCA).

Circulating levels of adipsin^43,44^ are inversely correlated with NASH regression in humans, and therefore, its downregulation fit with the robust improvement of liver histopathology observed in the present study. The Cell death-inducing DFF45-like effector (Cide) A and C gene (Fig. 4B,C) are known for their role in lipid droplets formation and triglycerides storage in hepatocytes, and we have previously shown that these genes are potently induced in the liver of mice feed a Western diet, while their expression is downregulated by BAR502^33,34,45^. In the present study we have confirmed this finding, validating Cide a and c as a potential target for BAR502. ElovL 5 (very long fatty acid elongase) elongates linoleic acid and α-linolenic acid to form arachidonic acid and eicosapentaenoic acid, respectively. Interestingly, we have found that both BAR502 alone or in combination with UDCA, reduced the expression of this elongase (Fig. 4D). Since, members of Elovl family contributes to obesity-induced insulin resistance, inhibition of Elovl5 might be beneficial in this model^46^.

Additional lipid-related genes, including lipoprotein lipase (Lpl), Perlipin (Plin)4, and Monoacylglycerol O-Acyltransferase (Mogat)1 that were modulated in this study are shown in Fig. 4E–L.

**Figure 5:**
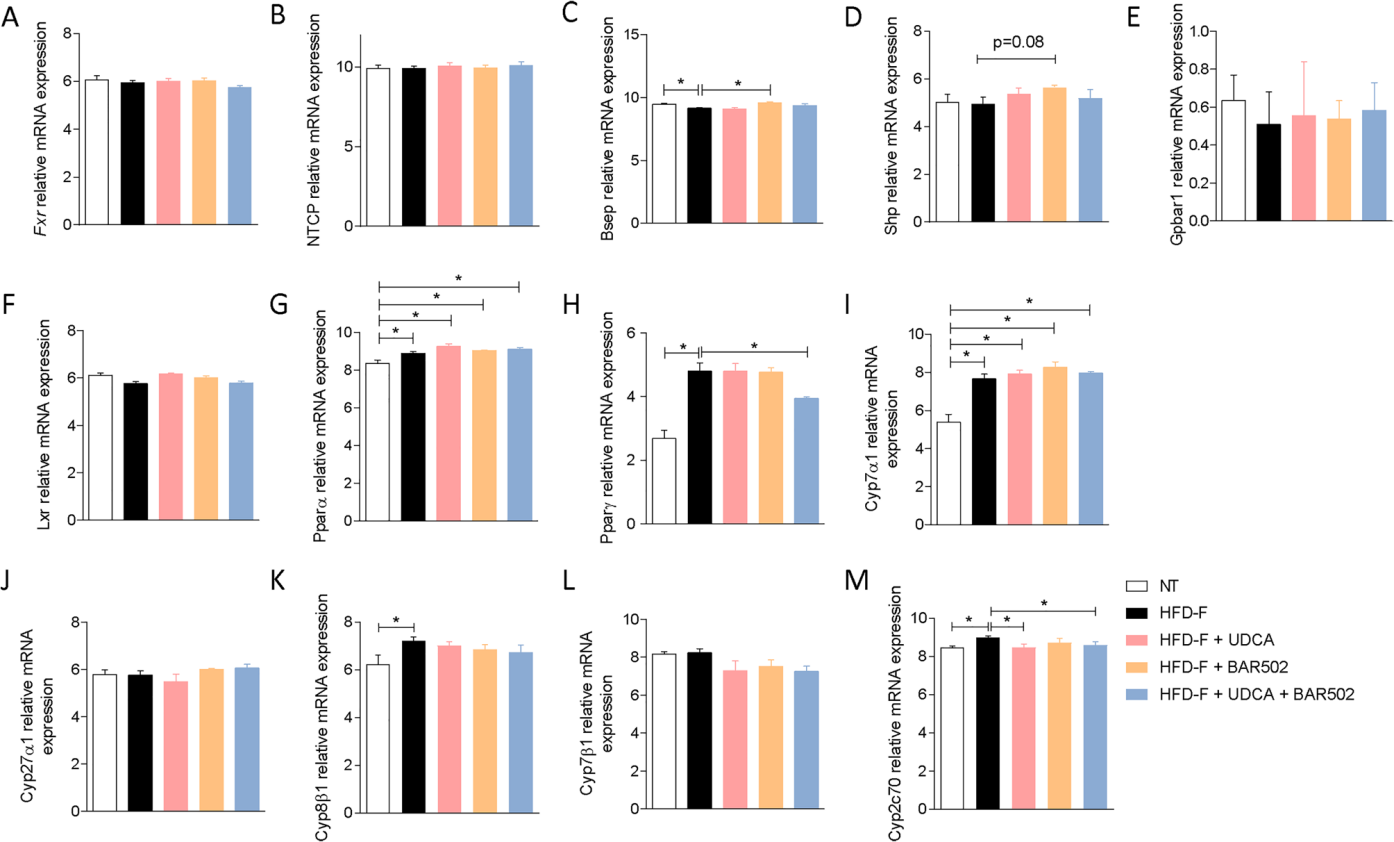
Hepatic expression of receptors and genes involved in bile acid synthesis. The expression levels of genes are extracted from RNA-seq analysis.

As shown in Fig. 5, exposure of mice to Western diet resulted in a dysfunction of liver FXR signalling. Indeed, while Fxr gene expression did not change in the liver of mice fed a western diet (Fig. 5A), expression of various FXR-regulated genes was modulated by the diet, including Bsep (downregulated), Cyp7α1 and Cyp8β1 (both upregulated) (Fig. 5B,I,K).

Along with the increase in the expression of Cyp2c70 (a gene in volved in the formation of MCAs) (Fig. 5M), these data suggest that exposure of mice to Western diet increases bile acid synthesis, including MCAs but partially impairs their excretion, which will increase the bile acids content in the liver (see Fig. 8). Treating mice with UDCA exerted no modulation on this patten (Fig. 5). In contrast treating mice with BAR502 alone or in combination partially modulated these changes: (1) BAR502 alone or in combination with UDCA increased the expression of Bsep and Shp (Fig. 5C,D), two FXR-regulated genes, but failed to reverse the expression of Cyp7α1 (Fig. 5I), a gene was expression is negatively regulated by FXR in a SHP-dependent and SHP-independent^19^ manner^25,47^. Together these data suggest: (a) the protective role of Cyp7α1 in cholesterol disposal in mice, and, (b) that negative regulation of Cyp7α1 by FXR is removed in the presence of a high cholesterol intake^12^. UDCA e BAR502 alone or in combination, reduced the expression of Cyp2c70 (Fig. 5M), suggesting a potential inhibition of MCAs synthesis^48^. In addition, we found that feeding mice a Western diet increased the expression of Pparα and γ (Fig. 5G,H).

### Effects of Bar502 and UDCA on adipose tissue and intestine

**Figure 6:**
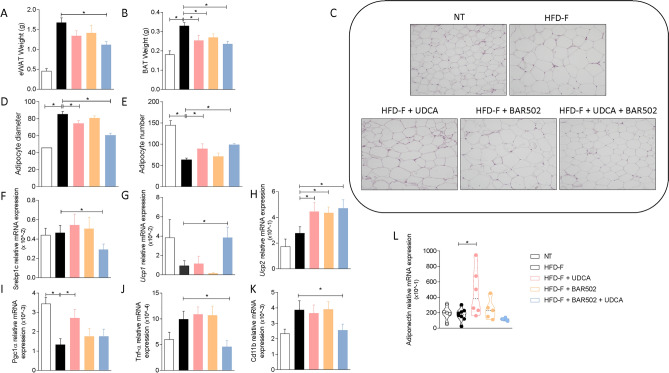
(**A**) Epididymal fat (eWAT) and (**B**) brown adipose tissue (BAT) weight, (**C**) H&E staining of eWAT tissues obtained at the end of the study. Original magnification × 40. (**D**) Adipocytes size and (**E**) number obtained from analysis of H&E staining performed with ImageJ. (**F**–**L**) Changes of mRNA expression of eWAT genes. The RT-PCR values were normalized against Gapdh. The data shown are the mean ± SE of 7–10 mice per group (*p < 0.05).

Because the WAT is not only the major storage tissue for fat but it also acts as an endocrine organ, we have then examined whether BAR502 and UDCA modulate fat depots in the model. Eating a high fat diet for 8 weeks increased the weight of both epWAT and BAT (Fig. 6A,B) as well as deposition of lipid droplets in the adipocytes resulting in adipocytes enlargement along with a decrease in their relative number (Fig. 6C–E).

Treating mice with BAR502 or UDCA alone or in combination, reversed some of these changes, and the combination of BAR5012 and UDCA significantly reduced fat deposition, as demonstrated by the reduced epWAt and BAT weights, along with reversal of adipocytes enlargement and number reduction (Fig. 6A–E). These phenotypic changes associated with changes in the expression of lipidogenic genes including Srebp1c whose expression was reduced in mice feed BAR502 and UDCA (Fig. 6F). Treating mice with BAR502 alone or in combination with UDCA modulated the expression of Ucp1, Ucp2 and Pgc1α^22,34^ (Fig. 6G-I). Additionally, the combination of Bar502 and UDCA, significantly reduced the expression of markers of inflammation such as Tnfα and Cd11 (Fig. 6J,K). Of relevance, UDCA increased the expression of adiponectin (Fig. 6L).

**Figure 7:**
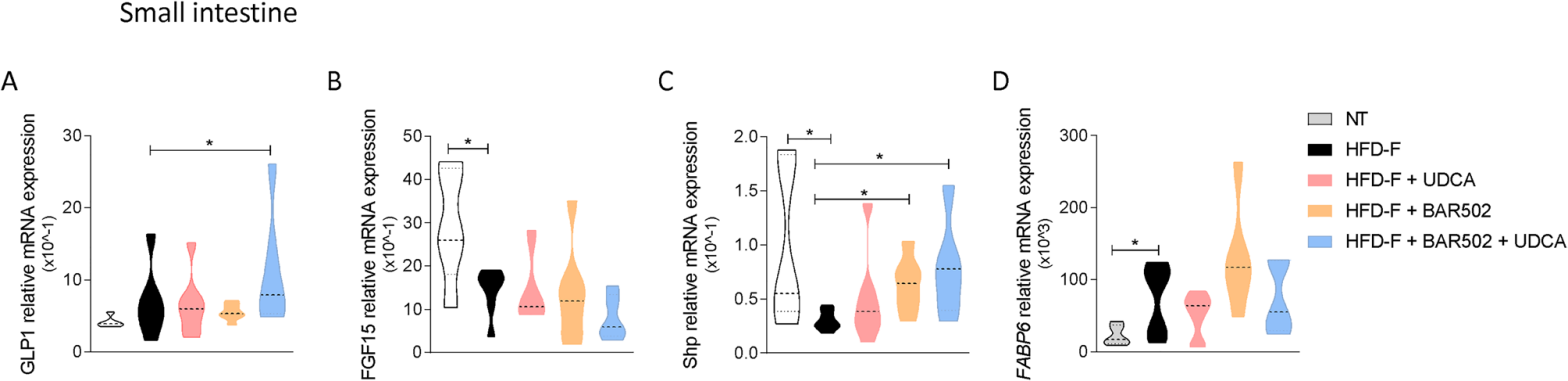
Relative mRNA expression of (**A**) pro-glucagon precursor, (**B**) FGF-15, (**C**) Shp and (**D**) FABP6 in small intestine samples. Data are normalized to Gapdh mRNA. Each result is the mean ± SEM of 7–11 mice per group. *p ≤ 0.05.

As shown in Fig. 7, feeding a WD also modulated the expression of several intestinal genes. The main changes were represented by an induction, thought it was not significant of Glp1 (Fig. 7A) and a robust increase of Fabp6 (also known as Ibabp)^19^ and a reduction of Fgf15 and Shp (Fig. 7B–D).

These changes were consistent with enhanced lipid and bile acid absorption (Fabp6), but also demonstrate that robust alteration of FXR signalling. Indeed, while reduction of Shp and Fgf15 signals a reduction of intestinal FXR, the induction of Fabp6, whose expression is positively regulated by SHP^49^, indicates that, as takes place in the liver, a high caloric intake supersede on physiological signals at multiple levels. Treating mice with UDCA and BAR502 alone or in combination, only partially restored these signals (Fig. 7B–D). Thus while the combination of the two agents increased the expression of Glp1 in the terminal ileum (a GPBAR1 regulated gene), BAR502 alone or in combination failed to increase the expression of intestinal Fgf15, despite a robust induction of intestinal Shp. This finding is consistent with the fact that liver expression of Cyp7a1 (a Fgf15 regulated gene)^19^ was not downregulated in mice feed with the combination of the two agents (Figs. 5 and 7).

### Bile acids composition

**Figure 8:**
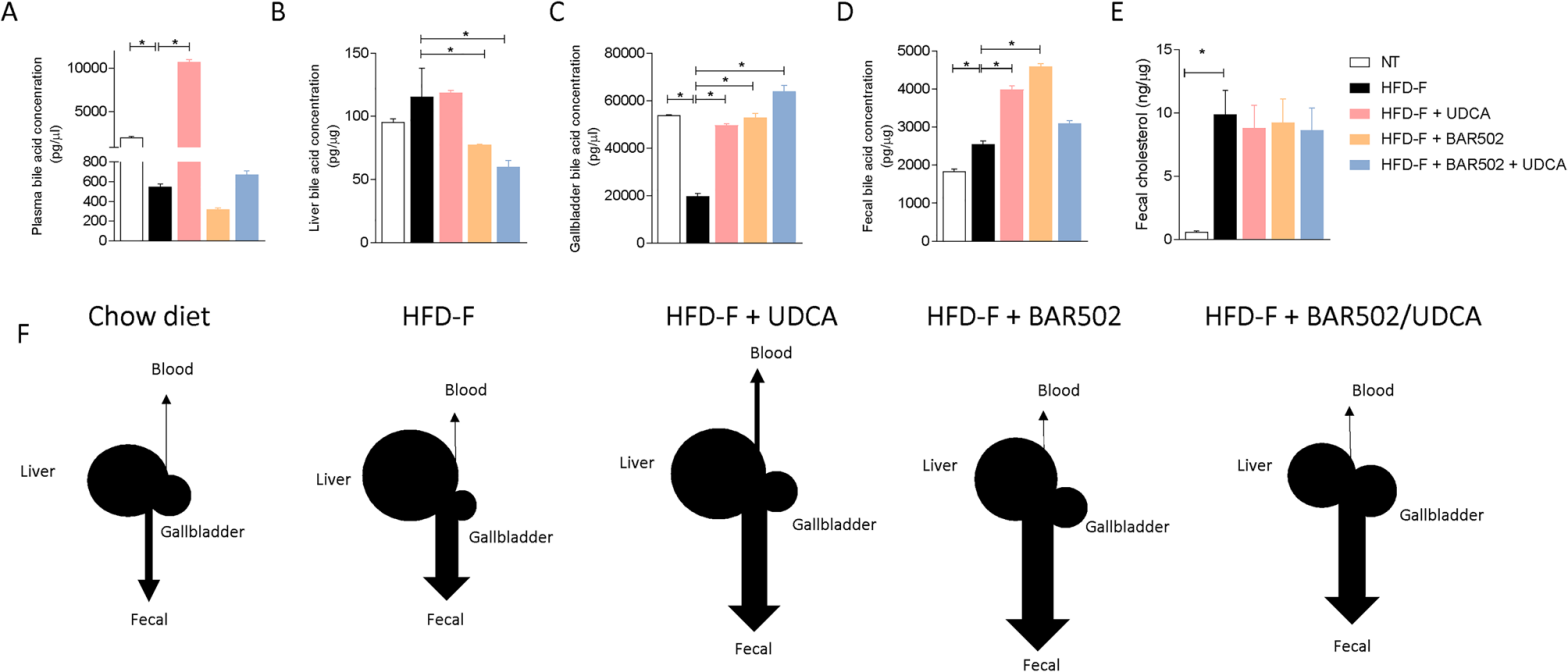
Bile acids concentration in: (**A**) Serum, (**B**) Liver, (**C**) Gallbladder and (**D**) Feces. (**E**) Cholesterol content in the stool. Each result is the mean ± SEM of 7–11 mice per group. *p ≤ 0.05. (**F**) Representative image of the bile acids content in cholesterol excretion in each experimental group.

Because an increased expression of Cyp7a1 and Cyp8b1 described in Fig. 4, would results in enhanced bile acid synthesis, we have then examined the composition of bile acid pool in the model and found that feeding a Western diet associated with a very robust expansion of the bile acid pool in the liver along with an increased fecal excretion of bile acids and cholesterol, while the plasma and gallbladder concentrations were reduced (Fig. 8A–E).

Treating mice with UDCA, BAR502 alone or in combination exerted multiple effects. Thus, while UDCA significantly increased serum, gallbladder and fecal bile acid concentrations in comparison to mice feed a Western diet alone (Fig. 8A–D,F), BAR502 reduced the liver bile acid concentrations while increased gallbladder and fecal excretion (Fig. 8). Interestingly the administration of BAR502 in combination with UDCA, reproduced some of the effects of BAR502 alone, since the combination therapy reduced liver bile acids concentrations and promoted their gallbladder accumulation but maintained the fecal excretion observed in mice feed a Western diet alone. These changes, together suggest that: (1) UDCA enhances serum bile acids and increases their excretion but has no impact on liver bile acids; (2) BAR502 alone, reduces bile acids in the liver because promotes their excretion in the gallbladder and feces, and 3) the combination of BAR502 and UDCA markedly reduce liver bile acid contents and promotes their excretion.

**Figure 9:**
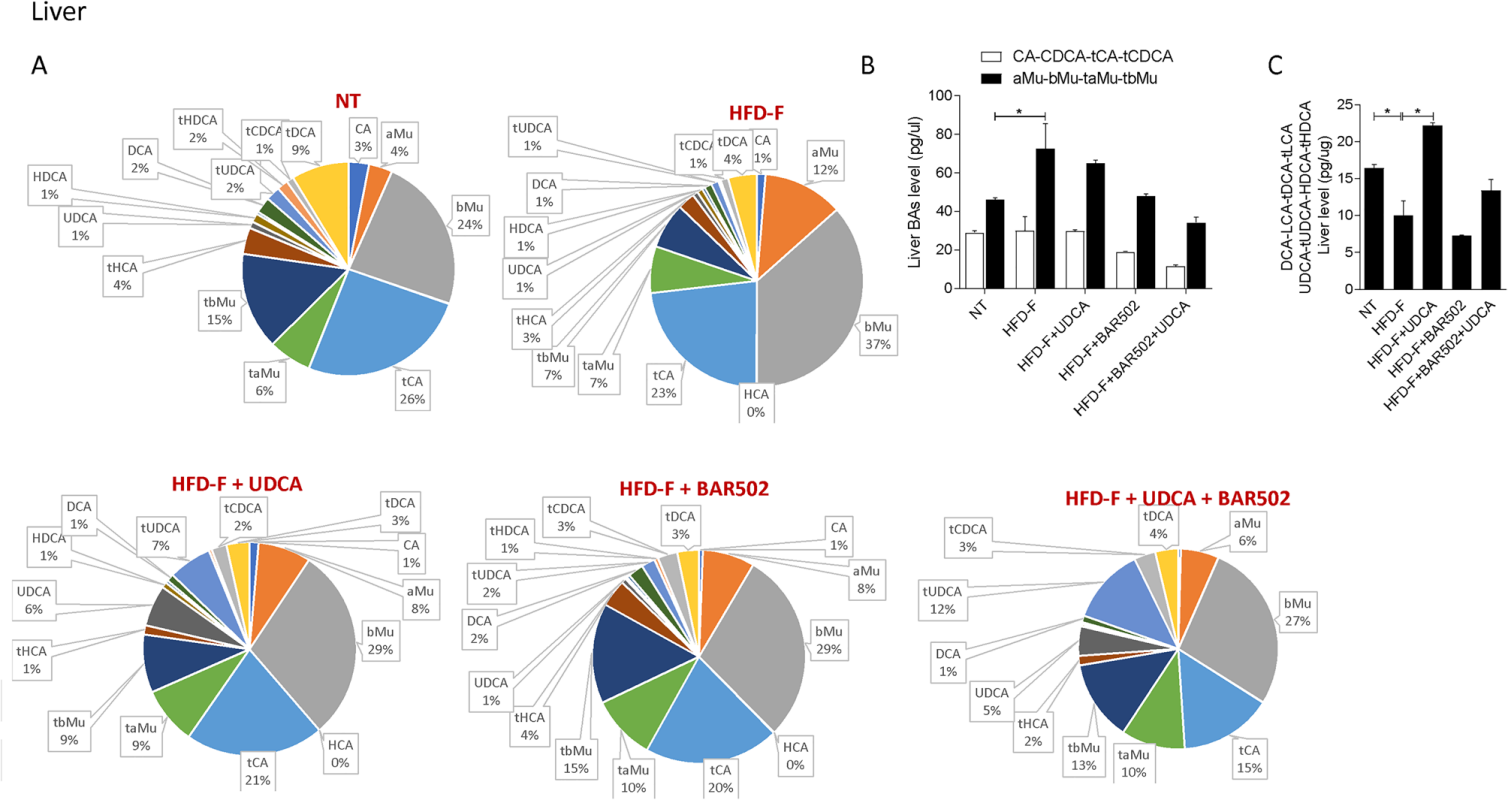
(**A**) Composition of liver bile acid pools. (**B**) Fxr agonist and antagonist bile acids content in liver. (**C**) Concentration of Gpbar1 agonist bile acids. Each result is the mean ± SEM of 7–11 mice per group. *p ≤ 0.05.

**Figure 10:**
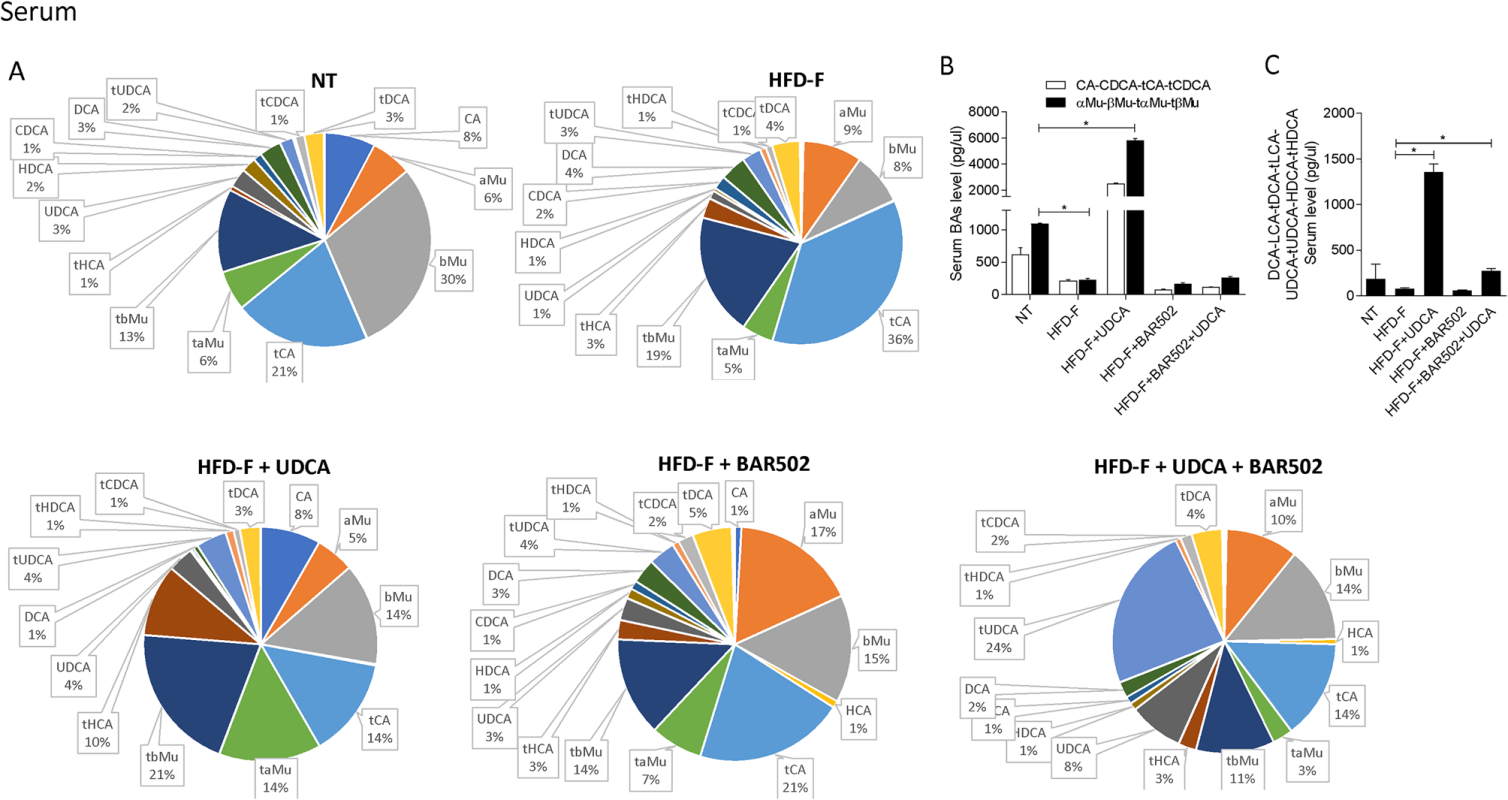
(**A**) Composition of Serum bile acid pools. (**B**) Fxr agonist and antagonist bile acids content in serum. (**C**) Concentration of Gpbar1 agonist bile acids. Each result is the mean ± SEM of 7–11 mice per group. *p ≤ 0.05.

**Figure 11:**
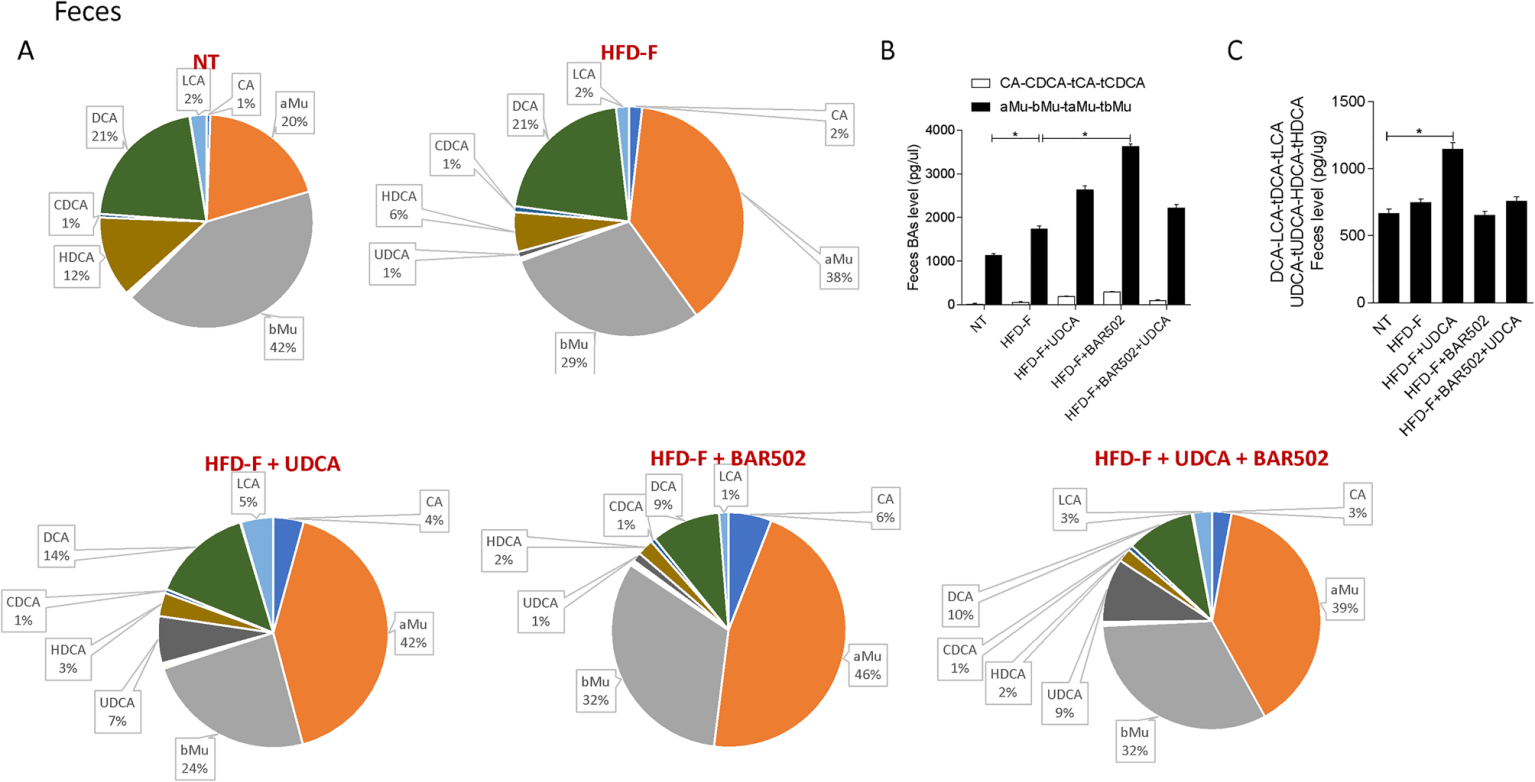
(**A**) Composition of fecal bile acid pools. (**B**) Fxr agonist and antagonist bile acids content in feces. (**C**) Concentration of Gpbar1 agonist bile acids. Each result is the mean ± SEM of 7–11 mice per group. *p ≤ 0.05.

**Figure 12:**
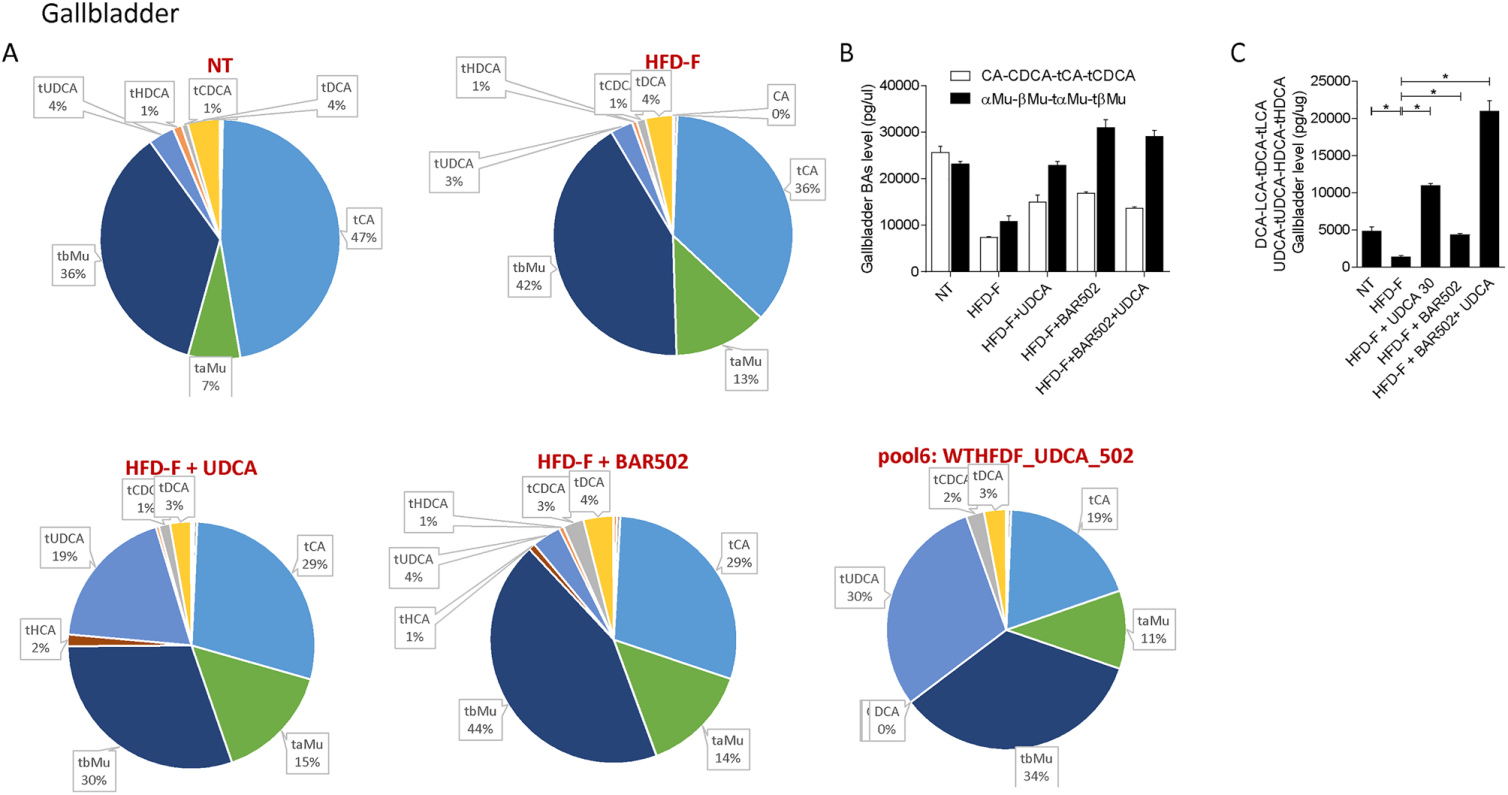
(**A**) Composition of gallbladder bile acid pools. (**B**) Fxr agonist and antagonist bile acids content in gallbladder. (**C**) Concentration of Gpbar1 agonist bile acids. Each result is the mean ± SEM of 7–11 mice per group. *p ≤ 0.05.

Qualitative analysis of bile acid composition, is shown in Figs. 9, 10, 11, 12.

The analysis of the various bile acid species demonstrates that exposure to high fat diet dramatically expands the content of MCAs in the liver (Fig. 9), while did the opposite in the serum (Fig. 10).

Since MCAs are FXR antagonists, it appears that exposure of mice to Western diet shifts the bile acid pool toward bile acid species, the TMCA, that are FXR antagonists deteriorating the FXR signaling in the liver (Fig. 9). A similar shift is observed in the feces (Fig. 11).

Together these data indicate that in mice feed a diet enriched in fat and cholesterol, FXR signaling is resettled to allow a conversion of cholesterol into bile acids which are then excreted with the feces (Fig. 8E). Treating mice with UDCA, dramatically increased serum bile acids concentrations (Fig. 8A). Importantly, while UDCA increased the concentrations of FXR agonists in the serum, it also increased the levels of those bile acids (UDCA, T-UDCA, DCA, LCA and HDCA) that are preferential agonists to GPBAR1 in serum, liver, gallbladder and intestine (Figs. 9, 10, 11, 12), suggesting a role for this receptor as a potential mediator of the effects exerted by UDCA. In contrast, BAR502 markedly enhanced the fecal excretion of those bile acid species that are FXR antagonists, including MCAs (Fig. 11), suggesting that this agent further inhibit FXR signaling in the intestine, while promotes GPBAR1 signaling in the enterohepatic circulation. The combination of the two agents, resulted in a significant increase of GPBAR1 agonists in the gallbladder (Fig. 12).

## Discussion

Liver metabolic diseases, encompassing to main clinical entities NAFLD and NASH, is a growing clinical problem contributing to the increasing burden of liver cancer worldwide^50^. While a large number of therapeutic approaches are currently under investigation for the treatment of NASH, the disease remains an un meet clinical needs^9^. NASH is a complex disease with multiple clinical subsets that might require targeted therapeutic approaches^51^. While development of single drug treatments has been the main strategy in the last decade, the fact that a large proportion of these approaches have failed to meet pre-specified endpoints^52^ strongly support the notion that treating NASH requires the development of combinatorial approaches. Building on this concept, we have now provided evidence that a combination of BAR502 and UDCA results in a profound remodeling of body’s lipid metabolism, restores insulin sensitivity and reverses histology features of steatohepatitis and hepatocytes ballooning and adipocytes inflammation.

BAR502, is dual GPBAR1/FXR ligand that is currently undergoing phase 1 evaluation^33,34^ (*ClinicalTrials.gov Identifier: NCT05203367*). Chemically, BAR502 is a non bile acid steroidal ligand for FXR, that is similarly to obeticholic acid (OCA), a FXR ligand clinically approved for the treatment of primary biliary cholangitis (PBC), is generated from the addition of an ethyl group in the position 6 to a steroid core, CDCA in the case of OCA^24,32^. Further on, in contrast to OCA, COOH group in the side chain of BAR502 is substituted by a OH group, a chemical substitution that prevents the its conjugation with glycine or taurine by hepatocytes^24,32^. Thus, in contrast to OCA that circulates as glycine and taurine salt and undergoes multiple cycles in the entero-hepatic circulation and accumulate in body, BAR502 is excreted with the urine and does not undergoes an entero-hepatic circulation^26,53^. In addition, BAR502 is dual FXR and GPBAR1 agonist^24,32^. In terms of FXR agonism, the EC50 of BAR502 in transactivation assays is ≈ 1 µM, that is higher than 300 nM we originally reported for 6-ECDCA (the original name of OCA) in 2002^24^, while the Ec50 for GPBAR1 is ≈ 900 nM^24,32^, making BAR502 almost equally potent toward the two receptors.

Several FXR agonists are currently developed for the treatment of NASH^29,54^. Cilofexor, tropifexor, nidufexor, MET 409, TERN101 and EYP001 have completed Phase II trials, while OCA has completed a phase III trial in patients with biopsy proven NASH^54^. While the results of these trials have shown various degree of efficacy in improving histopathology features (i.e. steatohepatitis, ballooning and fibrosis) of NASH and biochemical efficacy in reducing AST/ALT levels, none of these agents has been able to completely reverse NASH features, while side effects have emerged. OCA, the first in class of these agents, has shown efficacy in reducing liver inflammation and fibrosis in some Phase II and III trials but its use associates with a variety of side effects, the most common of which is pruritus (that is dose dependent and occurs in up to 50% in response to a dose of 25 mg/day) along with the worsening of a pro-atherogenic lipoprotein profile^28,55^. OCA has also been associated with severe liver decompensation, liver transplantation and death when administered in doses higher than 5 mg/week to cirrhotic PBC^27,53^. These effects seems to be class-dependent^56^, prompting the development of alternative approaches to NASH, that, however, have shown a certain lack of efficacy in various studies^57,58^.

Based on this background, we have designed a study to target multiple molecular pathways^54^, in addition to FXR, including GPBAR1, a receptor for secondary bile acids^59^. GPBAR1 is highly expressed in small intestine, adipose tissues, muscles and inflammatory cells, but not by hepatocytes^12^, and its activation in these tissues promotes the release of GLP-1^20^, ameliorates insulin sensitivity, increase energy expenditure by adipose tissues and promotes their transition from white to beige of brown phenotype^22,60^. Here, we have shown that BAR502 when administered alone at the dose of 30 mg/kg to mice feed a Western diet activates both FXR and GPBAR1 in target tissues and partially reverses biochemical and histopathology features caused by feeding a Western diet both in the liver and adipose tissue. However, these beneficial effects were partial, since BAR502 did not fully reversed steatohepatitis and hepatocytes ballooning, despite the fact that it reduced liver fibrosis and ameliorates the plasma lipid profile (LDL levels). These data are consistent with our previous results and confirm the therapeutic potential of BAR502^33^, but also advocate the possibility that combining BAR502 with other agents might improve its therapeutic potential.

UDCA is clinically approved bile acid^61^. UDCA has been shown effective in slowing disease progression in PBC patients, thus reducing the need for liver transplantation and increasing survival rate and quality of life^62^. In contrast, several clinical trials have reported that UDCA is only partially effective in reversing histopathology and biochemical features of NASH^38,39,63,64^. Furthermore, while using doses higher than 15 mg/kg (recommended for PBC) seems to expand the clinical efficacy of UDCA^39,64^ the lack of a formal trial prevents its use in the treatment of NASH. We have recently shown that UDCA is a weak GPBAR1 ligand^61^ and clinical studies have confirmed that UDCA increases GLP1 release form the intestine^63^ and there is evidence that UDCA ameliorates insulin sensitivity in NAFLD patients. We have confirmed these findings and demonstrated that UDCA exerts some beneficial effects in our model of NASH, but it failed to improve liver histopathology^63^ and did not reduced the neither hepatocytes ballooning or liver fibrosis.

The main result of the presents study has been the demonstration that a combination of BAR502 and UDCA achieve a substantial improvement in clinical efficacy in comparison to the two agents administered alone. Thus, not only the combination of the two agents reduced body weight gain by ≈ 10% but also effectively reversed biochemical and histopathology features of NASH. Thus, the combination of the two agents improved insulin sensitivity as assessed by OGTT, reduced AST/ALT and cholesterol, triglycerides, LDL and HDL plasma levels. Similarly, the combination of BAR502/UDCA significantly improved liver steatosis, inflammation, hepatocytes ballooning and fibrosis in the model. Lipid-enriched diets are extensively used to induce liver steatosis in murine models of NAFLD^13^. Nevertheless, mice are known for their resistance to develop the typical pro-atherogenic lipid profile observed in humans in response to chronic exposure to a high caloric intake and are protected against the development of vascular lesions that occurs in humans in these clinical setting^65^. Mice are a HDL-prone species, a pattern that associates with relatively low levels of VLDL and LDL^65^, a lipoprotein profile that in humans confers a lower risk to develop of cardiovascular disease^25,66^. The fact that a combination of BAR502 and UDCA improves the lipid profiles in this model might be, therefore, of translational relevance.

The beneficial effects exerted by the combination of BAR502 and UDCA on biochemical and histopathology features in the model, associates with a profound remodeling of liver transcriptome. Treating mice feed a western diet with combination of BAR502 and UDCA rewrote the expression of over 600 genes, a number significantly higher than the 273 remodeled by BAR502 alone and 473 that were modified by UDCA. Of relevance the three treatments shared the modulation of 43 genes only, highlighting the fact that each therapeutic regimen was acting on specific pathways. Among the genes that were potently modulated by the three treatments we detected Cidea, Elov5 and Mogat1^33,34^. These three genes are known for their role in regulating lipid metabolism. Low levels of CIDEA are associated in mice with increased thermogenesis and protects from obesity, while Mogat1 is a critical genes essential for triacylglycerol biosynthesis^45,46,67^.

In the search for a mechanism of action that could explain the beneficial effects exerts by the combination of the BAR502 and UDCA we have carried out a detailed analysis of the composition of bile acid pool. Conversion of cholesterol in to bile acids is a major mechanisms of cholesterol disposal in mice^48^. Thus, while in humans, only ≈ 10% of the total bile pool is disposed daily in feces and urine, this pathways is robustly amplified in mice exposed to a high fat diet^12^. The main bile acid biosynthetic pathway in both human and rodents is the classic pathway that accounts for at least 75% of the total bile pool, and in humans, produces: CDCA and CA^12^. In mice, CDCA is then converted into MCAs by Cyp2c70, generating a specie-specific bile acids profile^68^. In contrast to CA and CDCA, T-MCAs are FXR antagonists^69^. Since FXR regulates the expression/function of CYP7A1, the rate limiting enzyme of bile acid synthesis in the classic pathway, these specie-specificities have major consequences on the regulation of bile acid synthesis^70^. CYP7A1 expression expression/activity is negatively regulated by FXR in a SHP and FGF19 (FGF15 in mice) dependent manner^12^. SHP is a transcription factor that is directly regulated by FXR in hepatocytes^16^, while FGF19 is released from enterocytes under activation of FXR in the ileum^19^. In the presence of TMCAs, the braking signal exerted by FXR in the liver and ileum is partially removed, allowing an upregulation of CYP7A1 and an increased conversion of cholesterol into bile acids. A mechanism that can not take place in humans because primary bile acids in humans, CDCA and CA, represses CYP7A1 activity via FXR^16^.

 Results from our study suggest that the beneficial effects exerted by BAR502/UDCA might be due to their activity on bile acid metabolism. This view is supported by the following observations: (1) feeding mice a Western diet increases the liver expression of Cyp7a1, Cyp8b1 and Cyp2c70 mRNA and increases the liver synthesis of bile acids, while shifts the bile acid pool toward those bile acid species, αMCA, βMCA, T αMCA and βMCA that are FXR antagonists, while reduces the relative amounts of CA, CDCA and their taurine conjugates. (2) Mice cope with the Western diet by increasing the fecal excretion of bile acids and cholesterol^16^. These changes represent a protective adaptation to the high fat diet and are achieved by suppressing FXR activity in the liver and intestine. (3) Feeding a Western diet reduced the systemic levels of DCA and LCA, the two main endogenous GPBAR1 agonists^12^, suggesting a potential impairment of GPBAR1 signaling, that might contributes to the impairment of insulin sensitivity^25,66^. Together, these changes in the expression of liver bile acid synthetic pathways are likely due to reflect changes in the relative concentrations of MCAs, especially T-MCA, which are FXR antagonists in nature^69^. Treating mice with BAR502/UDCA resulted in several changes in bile acid pool composition, that could be summarized as follow: (1) BAR502/UDCA increased bile acid secretion from the liver as shown by decreased bile acid content in the liver and increased concentrations of bile acids in the gallbladder and feces; (2) in comparison to mice feed a Western diet, BAR502 and UDCA increased significantly the systemic concentrations of those bile acids, LCA, DCA, UDCA and HyoDCA that are GPBAR1 ligands (Figs. 8 and 9), suggesting that these changes might contribute the beneficial effects the BAR502/UDCA exert on insulin sensitivity; (3) a similar shift toward bile acids that are GPBAR1 agonists was detected in the liver, which might contribute to the anti-inflammatory and anti-fibrotic activity observed in mice treated with Bar502/UDCA. In fact, not only GPBAR1 is expressed on liver macrophages and activated HSC, but its activation promotes a counter-inflammatory phenotype by Kupffer cells and macrophages^71,72^. These findings are consistent with previous investigations showing that GPBAR1 activation mediates the beneficial effects of sleeve gastrectomy, a widely used of bariatric surgery, in mouse models NASH^73,74^. These findings might, therefore have translational relevance, since bariatric surgeries are increasingly used to treat obesity-related NASH, and animal models suggest that one of the putative beneficial effects of the sleeve gastrectomy is a reduction of bile acid pool in a FXR independent manner^75^.

One additional beneficial effect induced BAR502 and UDCA in this model, was the modulation of adipose tissue. Indeed, while the two agents exerted some beneficial effects on the WAT and BAT, the combination of the two reduced the weight of WAT and BAT, prevented the increase of adipocytes size and increased the number of adipocytes. Together with the fact that the combination of BAR502/UDCA, increased the WAT’s expression of Ucp1 and Ucp2 while negatively regulated the expression of Tnfα and Cd11b, these results support the view that part of the beneficial effects manifested in mice administered BAR502/UDCA were due to a general improvement of adipose tissues health^76^.

In conclusion we have shown that in a mouse model of NAFLD/NASH administration of an investigational new drug, BAR502, in combination with UDCA, exerts beneficia effects that were superior to that exerted by each agent individually. The combination of the two agents reverses almost completely the liver features of NASH, including hepatocytes ballooning, promoted a shift of bile acid pool toward GPBAR1 biased bile acid species and reversed the pro-atherogenic lipid profile that develop feeding a Western diet. These results support the clinical development of BAR502 in combination with UDCA.

## Materials and methods

### Chemicals

UDCA was provided by ICE SpA (Reggio Emilia, Italy). BAR502 was prepared by Prof. Angela Zampella. The agents were dissolved daily in 1% methyl cellulose before administration by gavage (100 μl).

### Animal model

C57BL/6J male mice were fed a high fat diet (HFD) containing 59 kJ% fat plus 1% cholesterol, w/o sugar (ssniff^®^ EF R/M acc. D12330 mod. 22,7 ME/Kg) and fructose (HFD-F) in drinking water (42 g/l), or normal chow diet (standard diet Mmucedola S.r.l. 4RF21: 18,5% proteins, 53,5% carbohydrates, 3% Fats) for 8 weeks. Food intake was estimated as the difference of weight between the offered and the remnant amount of food at 3-days intervals. The food was provided as pressed pellets and the residual spillage was not considered. After 10 days, HFD-F mice were randomized to receive HFD-F alone or BAR502 (30 mg/kg/day) UDCA (30 mg/kg/day) or the combination of the two treatments (BAR502-UDCA) by gavage for 7 weeks. Mice were housed under controlled temperature (22 °C) and photoperiods (12:12-h light/dark cycle), allowed unrestricted access to standard mouse chow and tap water. The general health of the animals was monitored daily by the Veterinarian in the animal facility. At the day of sacrifice, fed mice were deeply anesthetized with a mixture of tiletamine hydrochloride and zolazepam hypochloride/xylazine at a dose of 50/5 mg/kg and sacrificed before 12 PM.

### Anthropometrical determinations

The abdominal circumference (AC) (immediately anterior to the forefoot), body weight and body length (nose-to-anus or nose–anus length), were measured in anaesthetized mice at time of sacrifice. The body weight and body length were used to calculate the Lee index (= cube root of body weight (g)/nose-to-anus length (cm)).

### Biochemical analyses and OGTT

Cholesterol, triglycerides, HDL and LDL plasmatic levels were quantified using an automated clinical chemistry analyzer (Cobas, Roche Basel, Switzerland). After 7 weeks of HFD-F mice were fasted overnight and orally administered glucose (1.5 g/kg body weight) for OGTT. The blood glucose concentrations were measured at 0, 15, 30, 60, 90, and 120 min after feeding or injection using a portable glucose meter (Accu-Check Go, Roche Basel, Switzerland).

### Bile acid determinations

Gallbladder, liver, serum and fecal bile acid concentrations were measured by a liquid chromatography-tandem mass spectrometry (MS/MS), as detailed elsewhere^25,63^.

### Histopathology

For histological examination, portions of the right and left liver lobes were fixed in 10% formalin, embedded in paraffin, sectioned and stained with Sirius red and/or Hematoxylin/Eosin (H&E), for morphometric analysis. NASH severity (steatosis, hepatocytes ballooning, lobular inflammation and portal inflammation) was scored in H&E-stained cross sections using an adapted grading system of human NASH^77^.

Hepatic fibrosis score was evaluated in Sirius red stained sections using an adapted grading system of human NASH. The presence of pathological collagen deposition was scored as either absent (0), observed within perisinusoidal/perivenular or periportal area (1), within both perisinusoidal and periportal areas (2), bridging fibrosis (3) or cirrhosis (4). The sum of the scores (degree of steatosis, hepatocyte ballooning, lobular inflammation, and portal inflammation) was considered as the total pathology score.

### Reverse transcription of mRNA and Real-Time PCR

RNA was extracted from liver, epididymal adipose tissue (eWAT) and small intestine using TRI reagent (Zymo Research) and Direct-zol™ RNA MiniPrep w/Zymo-Spin™ IIC Columns (Zymo Research, Irvine, CA). RNA from small intestine and eWAT sample was reverse transcribed using Fastgene Scriptase basic cDNA kit (Nippon Genetics Europe GmbH, Düren, DE); 10 ng of cDNA was amplified in a 20-μl solution containing 200 nM each primer and 10 μl of PowerUp SYBR Master Mix (Thermo Fisher Scientific, Waltham, MA). All reactions were performed in triplicate using the following thermal cycling conditions: 3 min at 95 °C, followed by 40 cycles of 95 °C for 15 s, 56 °C for 20 s, and 72 °C for 30 s, using a StepOnePlus system (Applied Biosystems, Foster City, CA). The relative mRNA expression was calculated accordingly to the ΔCt method. Primers were designed using the software PRIMER3 (http://frodo.wi.mit.edu/primer3/) using published data obtained from the NCBI database. The primers used were as following (forward and reverse):

mGapdh (for CTGAGTATGTCGTGGAGTCTAC; rev GTTGGTGGTGCAGGATGCATTG), mTNFα (for CCACCACGCTCTTCTGTCTA; rev AGGGTCTGGGCCATAGAACT), mCD11b (for AAGGATTCAGCAAGCCAGAA; rev TAGCAGGAAAGATGGGATGG), mPgc-1α (for CTTAGCACTCAGAACCATGCAG; rev AATGCTCTTCGCTTTATTGCTC), mSrebp1c (for GATCAAAGAGGAGCCAGTGC; rev TAGATGGTGGCTGCTGAGTG), mAdiponectin (for ATGCAGGTCTTCTTGGTCCT; rev GAGCGATACACATAAGCGGC), mUcp2 (for TTGCCCGTAATGCCATTGTC; rev GCAAGGGAGGTCATCTGTCA), mUcp1 (for TCCATGTACACCAAGGAAGGA; rev TCAGCTGTTCAAAGCACACA), mFgf15 (for GCTCTGAAGACGATTGCCAT; rev ACAGTCCATTTCCTCCCTGA) mGlp1 (for CCCCAGATCATTCCCAGCTT; rev CGGGAGTCCAGGTATTTGCT), Shp (for ACGATCCTCTTCAACCCAGA; rev AGGGCTCCAAGACTTCACAC) and Fabp6 (for CACCATTGGCAAAGAATGTG; rev CCGAAGTCTGGTGATAGTTGG).

### AmpliSeq transcriptome

From liver RNA, libraries were generated using the Ion AmpliSeq™ Transcriptome Mouse Gene Expression Core Panel and Chef-Ready Kit (Comprehensive evaluation of AmpliSeq transcriptome, a whole transcriptome RNA sequencing methodology) (Thermo Fisher Scientific, Waltham, MA). Template-Positive Ion Sphere™ Particles (Thermo Fisher Scientific, Waltham, MA) were loaded on Ion 540™ Chips, using the Ion 540™ Kit-Chef (Thermo Fisher Scientific, Waltham, MA). Sequencing was performed on an Ion S5™ Sequencer with Torrent Suite™ Software v6 (Thermo Fisher Scientific, Waltham, MA). The analyses were performed as previously described^25^.

### Statistical analysis

All of the data are shown as the means ± SEM. Difference among groups was estimated using one-way ANOVA followed by Tukey’s post hoc test, or by the *t* test analysis, or by two-way ANOVA followed by Bonferroni’s post hoc test when appropriated (GraphPad Prism 5.0 software). Significance was set up at p < 0.05.

### Ethics approval

The experimental protocols were approved by the Animal Care and Use Committee of the University of Perugia and by the Italian Minister of Health and Istituto Superiore di Sanità (Italy) and were in agreement with the European guidelines for use of experimental animals (permission n. 583/2017-PR). The study is reported in accordance with ARRIVE guidelines.

## Supplementary Information

Supplementary Tables.

## Data Availability

The datasets generated during the current study are available in the Mendeley data repository (Mendeley Data, 10.17632/bs4hshm249.1).

## Abbreviations

NAFLD: Non-alcoholic fatty liver disease
NASH: Non-alcoholic steatohepatitis
FXR: Farnesoid X receptor
GPBAR1: G protein bile acid receptor 1
OCA: Obeticholic acid
CVD: Cardiovascular disease
CYP7A1: Cholesterol 7α-hydroxylase
CYP2C70: Cytochrome P450 family 2 subfamily *c* polypeptide 70
CDCA: Chenodeoxycholic acid
CA: Cholic acid
DCA: Deoxycholic acid
LCA: Lithocholic acid
HYO-DCA: Hyo-deoxycholic acid
MCA: Muricholic acid
eWAT: Epididymal white adipose tissue
BAT: Brow adipose tissue
PPARα: Peroxisome-proliferator activated receptor alpha
PPARγ: Peroxisome-proliferator activated receptor gamma

## References

[1] Rinella ME, Sanyal AJ (2016). Management of NAFLD: A stage-based approach. Nat. Rev. Gastroenterol. Hepatol..

[2] Sherif ZA, Saeed A, Ghavimi S, Nouraie S-M, Laiyemo AO, Brim H, Ashktorab H (2016). Global epidemiology of nonalcoholic fatty liver disease and perspectives on US minority populations. Dig. Dis. Sci..

[3] Younossi Z, Anstee QM, Marietti M, Hardy T, Henry L, Eslam M, George J, Bugianesi E (2018). Global burden of NAFLD and NASH: Trends, predictions, risk factors and prevention. Nat. Rev. Gastroenterol. Hepatol..

[4] Dowman JK, Tomlinson JW, Newsome PN (2010). Pathogenesis of non-alcoholic fatty liver disease. QJM.

[5] Jiao N, Baker SS, Chapa-Rodriguez A, Liu W, Nugent CA, Tsompana M, Mastrandrea L, Buck MJ, Baker RD, Genco RJ, Zhu R, Zhu L (2018). Suppressed hepatic bile acid signalling despite elevated production of primary and secondary bile acids in NAFLD. Gut.

[6] Masuoka HC, Chalasani N (2013). Nonalcoholic fatty liver disease: An emerging threat to obese and diabetic individuals. Ann. N. Y. Acad. Sci..

[7] Armstrong MJ, Adams LA, Canbay A, Syn WK (2014). Extrahepatic complications of nonalcoholic fatty liver disease. Hepatology.

[8] Hung CK, Bodenheimer HCJ (2018). Current treatment of nonalcoholic fatty liver disease/nonalcoholic steatohepatitis. Clin. Liver Dis..

[9] Fiorucci S, Biagioli M, Distrutti E (2018). Future trends in the treatment of non-alcoholic steatohepatitis. Pharmacol. Res..

[10] Russell DW (2003). The enzymes, regulation, and genetics of bile acid synthesis. Annu. Rev. Biochem..

[11] Chiang JYL, Ferrell JM (2018). Bile acid metabolism in liver pathobiology. Gene Expr..

[12] Fiorucci S, Distrutti E, Carino A, Zampella A, Biagioli M (2021). Bile acids and their receptors in metabolic disorders. Prog. Lipid Res..

[13] Eng JM, Estall JL (2021). Diet-induced models of non-alcoholic fatty liver disease: Food for thought on sugar, fat, and cholesterol. Cells.

[14] Ferrell JM, Pathak P, Boehme S, Gilliland T, Chiang JYL (2019). Deficiency of both Farnesoid X receptor and Takeda g protein-coupled receptor 5 exacerbated liver fibrosis in mice. Hepatology.

[15] Chiang JYL (2009). Bile acids: Regulation of synthesis. J. Lipid Res..

[16] Goodwin B, Jones SA, Price RR, Watson MA, McKee DD, Moore LB, Galardi C, Wilson JG, Lewis MC, Roth ME, Maloney PR, Willson TM, Kliewer SA (2000). A regulatory cascade of the nuclear receptors FXR, SHP-1, and LRH-1 represses bile acid biosynthesis. Mol. Cell.

[17] Seol W, Choi HS, Moore DD (1996). An orphan nuclear hormone receptor that lacks a DNA binding domain and heterodimerizes with other receptors. Science.

[18] Song KH, Li T, Owsley E, Strom S, Chiang JY (2009). Bile acids activate fibroblast growth factor 19 signaling in human hepatocytes to inhibit cholesterol 7alpha-hydroxylase gene expression. Hepatology.

[19] Inagaki T, Choi M, Moschetta A, Peng L, Cummins CL, McDonald JG, Luo G, Jones SA, Goodwin B, Richardson JA, Gerard RD, Repa JJ, Mangelsdorf DJ, Kliewer SA (2005). Fibroblast growth factor 15 functions as an enterohepatic signal to regulate bile acid homeostasis. Cell Metab..

[20] Maruyama T, Miyamoto Y, Nakamura TT, Tamai Y, Okada H, Sugiyama E, Itadani H, Tanaka K, Nakamura TT, Itadani H, Tanaka K (2002). Identification of membrane-type receptor for bile acids (M-BAR). Biochem. Biophys. Res. Commun..

[21] Kawamata Y, Fujii R, Hosoya M, Harada M, Yoshida H, Miwa M, Fukusumi S, Habata Y, Itoh T, Shintani Y, Hinuma S, Fujisawa Y, Fujino M (2003). A G protein-coupled receptor responsive to bile acids. J. Biol. Chem..

[22] Watanabe M, Houten SM, Mataki C, Christoffolete MA, Kim BW, Sato H, Messaddeq N, Harney JW, Ezaki O, Kodama T, Schoonjans K, Bianco AC, Auwerx J (2006). Bile acids induce energy expenditure by promoting intracellular thyroid hormone activation. Nature.

[23] Scheja L, Heeren J (2016). Metabolic interplay between white, beige, brown adipocytes and the liver. J. Hepatol..

[24] Pellicciari R, Fiorucci S, Camaioni E, Clerici C, Costantino G, Maloney PRR, Morelli A, Parks DJJ, Willson TMM (2002). 6alpha-ethyl-chenodeoxycholic acid (6-ECDCA), a potent and selective FXR agonist endowed with anticholestatic activity. J. Med. Chem..

[25] Marchianò S, Biagioli M, Roselli R, Zampella A, Di Giorgio C, Bordoni M, Bellini R, Morretta E, Monti MC, Distrutti E, Fiorucci S (2022). Atorvastatin protects against liver and vascular damage in a model of diet induced steatohepatitis by resetting FXR and GPBAR1 signaling. FASEB J. Off. Publ. Fed. Am. Soc. Exp. Biol..

[26] Fiorucci S, Di Giorgio C, Distrutti E (2019). Obeticholic acid: An update of its pharmacological activities in liver disorders. Handb. Exp. Pharmacol..

[27] Carino A, Biagioli M, Marchianò S, Fiorucci C, Bordoni M, Roselli R, Di Giorgio C, Baldoni M, Ricci P, Monti MCC, Morretta E, Zampella A, Distrutti E, Fiorucci S (2020). Opposite effects of the FXR agonist obeticholic acid on Mafg and Nrf2 mediate the development of acute liver injury in rodent models of cholestasis. Biochim. Biophys. Acta Mol. Cell Biol. Lipids.

[28] Neuschwander-Tetri BA, Loomba R, Sanyal AJ, Lavine JE, Van Natta ML, Abdelmalek MF, Chalasani N, Dasarathy S, Diehl AM, Hameed B, Kowdley KV, McCullough A, Terrault N, Clark JM, Tonascia J, Brunt EM, Kleiner DE, Doo E, Network NCR (2015). Farnesoid X nuclear receptor ligand obeticholic acid for non-cirrhotic, non-alcoholic steatohepatitis (FLINT): A multicentre, randomised, placebo-controlled trial. Lancet.

[29] Fiorucci S, Biagioli M, Sepe V, Zampella A, Distrutti E (2020). Bile acid modulators for the treatment of nonalcoholic steatohepatitis (NASH). Expert Opin. Investig. Drugs.

[30] Trauner M, Gulamhusein A, Hameed B, Caldwell S, Shiffman ML, Landis C, Eksteen B, Agarwal K, Muir A, Rushbrook S, Lu X, Xu J, Chuang JC, Billin AN, Li G, Chung C, Subramanian GM, Myers RP, Bowlus CL, Kowdley KV (2019). The nonsteroidal farnesoid X receptor agonist cilofexor (gs-9674) improves markers of cholestasis and liver injury in patients with primary sclerosing cholangitis. Hepatology.

[31] Chianelli D, Rucker PV, Roland J, Tully DC, Nelson J, Liu X, Bursulaya B, Hernandez ED, Wu J, Prashad M, Schlama T, Liu Y, Chu A, Schmeits J, Huang DJ, Hill R, Bao D, Zoll J, Kim Y, Groessl T, McNamara P, Liu B, Richmond W, Sancho-Martinez I, Phimister A, Seidel HM, Badman MK, Joseph SB, Laffitte B, Molteni V (2020). Nidufexor (LMB763), a novel FXR modulator for the treatment of nonalcoholic steatohepatitis. J. Med. Chem..

[32] D’Amore C, Di Leva FSS, Sepe V, Renga B, Del Gaudio C, D’Auria MVV, Zampella A, Fiorucci S, Limongelli V (2014). Design, synthesis, and biological evaluation of potent dual agonists of nuclear and membrane bile acid receptors. J. Med. Chem..

[33] Carino A, Marchianò S, Biagioli M, Fiorucci C, Zampella A, Monti MCC, Morretta E, Bordoni M, Di Giorgio C, Roselli R, Distrutti E, Fiorucci S, Ricci P, Distrutti E, Fiorucci S (2019). Transcriptome analysis of dual FXR and GPBAR1 agonism in rodent model of NASH reveals modulation of lipid droplets formation. Nutrients.

[34] Carino A, Cipriani S, Marchianò S, Biagioli M, Santorelli C, Donini A, Zampella A, Monti MCC, Fiorucci S (2017). BAR502, a dual FXR and GPBAR1 agonist, promotes browning of white adipose tissue and reverses liver steatosis and fibrosis. Sci. Rep..

[35] Lindor K (2007). Ursodeoxycholic acid for the treatment of primary biliary cirrhosis. N. Engl. J. Med..

[36] Xiang Z, Chen Y, Ma K, Ye Y, Zheng L, Yang Y, Li Y, Jin X (2013). The role of ursodeoxycholic acid in non-alcoholic steatohepatitis: A systematic review. BMC Gastroenterol..

[37] Troisi G, Crisciotti F, Gianturco V, D’Ottavio E, Lo Iacono C, Formosa V, Bernardini S, Bellomo A, Marigliano B, Marigliano V (2013). The treatment with ursodeoxycholic acid in elderly patients affected by NAFLD and metabolic syndrome: A case–control study. Clin. Ter..

[38] Lindor KD, Kowdley KV, Heathcote EJ, Harrison ME, Jorgensen R, Angulo P, Lymp JF, Burgart L, Colin P (2004). Ursodeoxycholic acid for treatment of nonalcoholic steatohepatitis: Results of a randomized trial. Hepatology.

[39] Leuschner UFH, Lindenthal B, Herrmann G, Arnold JC, Rössle M, Cordes H-J, Zeuzem S, Hein J, Berg T (2010). High-dose ursodeoxycholic acid therapy for nonalcoholic steatohepatitis: A double-blind, randomized, placebo-controlled trial. Hepatology.

[40] Arab JP, Karpen SJ, Dawson PA, Arrese M, Trauner M (2017). Bile acids and nonalcoholic fatty liver disease: Molecular insights and therapeutic perspectives. Hepatology.

[41] Siddiqui MS, Van Natta ML, Connelly MA, Vuppalanchi R, Neuschwander-Tetri BA, Tonascia J, Guy C, Loomba R, Dasarathy S, Wattacheril J, Chalasani N, Sanyal AJ, NASH CRN (2020). Impact of obeticholic acid on the lipoprotein profile in patients with non-alcoholic steatohepatitis. J. Hepatol..

[42] Yu C, Chen J, Ma J, Zang L, Dong F, Sun J, Zheng M (2020). Identification of key genes and signaling pathways associated with the progression of gastric cancer. Pathol. Oncol. Res..

[43] Gu Y, Luo J, Chen Q, Qiu Y, Zhou Y, Wang X, Qian X, Liu Y, Xie J, Xu Z, Ling W, Chen Y, Yang L (2022). Inverse association of serum adipsin with the remission of nonalcoholic fatty-liver disease: A 3-year community-based cohort study. Ann. Nutr. Metab..

[44] Qiu Y, Wang S-F, Yu C, Chen Q, Jiang R, Pei L, Huang Y-L, Pang N-Z, Zhang Z, Ling W, Yang L (2019). Association of circulating adipsin, visfatin, and adiponectin with nonalcoholic fatty liver disease in adults: A case–control study. Ann. Nutr. Metab..

[45] Xu W, Wu L, Yu M, Chen F-J, Arshad M, Xia X, Ren H, Yu J, Xu L, Xu D, Li JZ, Li P, Zhou L (2016). Differential roles of cell death-inducing DNA fragmentation factor-α-like effector (CIDE) proteins in promoting lipid droplet fusion and growth in subpopulations of hepatocytes. J. Biol. Chem..

[46] Matsuzaka T, Kuba M, Koyasu S, Yamamoto Y, Motomura K, Arulmozhiraja S, Ohno H, Sharma R, Shimura T, Okajima Y, Han S-I, Aita Y, Mizunoe Y, Osaki Y, Iwasaki H, Yatoh S, Suzuki H, Sone H, Takeuchi Y, Yahagi N, Miyamoto T, Sekiya M, Nakagawa Y, Ema M, Takahashi S, Tokiwa H, Shimano H (2020). Hepatocyte ELOVL fatty acid elongase 6 determines ceramide acyl-chain length and hepatic insulin sensitivity in mice. Hepatology.

[47] Huang Y, Li W, Dong L, Li R, Wu Y (2013). Effect of statin therapy on the progression of common carotid artery intima-media thickness: An updated systematic review and meta-analysis of randomized controlled trials. J. Atheroscler. Thromb..

[48] Guo GL, Chiang JYL (2020). Is CYP2C70 the key to new mouse models to understand bile acids in humans?. J. Lipid Res..

[49] Ferrebee CB, Li J, Haywood J, Pachura K, Robinson BS, Hinrichs BH, Jones RM, Rao A, Dawson PA (2018). Organic solute transporter α-β protects ileal enterocytes from bile acid-induced injury. Cell. Mol. Gastroenterol. Hepatol..

[50] Tan DJH, Setiawan VW, Ng CH, Lim WH, Muthiah MD, Tan EX, Dan YY, Roberts LR, Loomba R, Huang DQ (2022). Global burden of liver cancer in males and females: Changing etiological basis and the growing contribution of NASH. Hepatology.

[51] Taylor RJRS, Taylor RJRS, Bayliss S, Hagström H, Nasr P, Schattenberg JM, Ishigami M, Toyoda H, Wai-Sun Wong V, Peleg N, Shlomai A, Sebastiani G, Seko Y, Bhala N, Younossi ZM, Anstee QM, McPherson S, Newsome PN (2020). Association between fibrosis stage and outcomes of patients with nonalcoholic fatty liver disease: A systematic review and meta-analysis. Gastroenterology.

[52] Ratziu V, Friedman SL (2020). Why do so many NASH trials fail?. Gastroenterology.

[53] Fiorucci S, Distrutti E (2019). The pharmacology of bile acids and their receptors. Handb. Exp. Pharmacol..

[54] Ratziu V, Francque S, Sanyal A (2022). Breakthroughs in therapies for NASH and remaining challenges. J. Hepatol..

[55] Rinella ME, Dufour J-F, Anstee QM, Goodman Z, Younossi Z, Harrison SA, Loomba R, Sanyal AJ, Bonacci M, Trylesinski A, Natha M, Shringarpure R, Granston T, Venugopal A, Ratziu V (2022). Non-invasive evaluation of response to obeticholic acid in patients with NASH: Results from the REGENERATE study. J. Hepatol..

[56] Venetsanaki V, Karabouta Z, Polyzos SA (2019). Farnesoid X nuclear receptor agonists for the treatment of nonalcoholic steatohepatitis. Eur. J. Pharmacol..

[57] Shen B, Lu LG (2021). Efficacy and safety of drugs for nonalcoholic steatohepatitis. J. Dig. Dis..

[58] Ratziu V, Sanyal A, Harrison SA, Wong VW-S, Francque S, Goodman Z, Aithal GP, Kowdley KV, Seyedkazemi S, Fischer L, Loomba R, Abdelmalek MF, Tacke F (2020). Cenicriviroc treatment for adults with nonalcoholic steatohepatitis and fibrosis: Final analysis of the phase 2b CENTAUR study. Hepatology.

[59] Keitel V, Stindt J, Häussinger D (2019). Bile acid-activated receptors: GPBAR1 (TGR5) and other G protein-coupled receptors. Handb. Exp. Pharmacol..

[60] Carino A, Cipriani S, Marchianò S, Biagioli M, Scarpelli P, Zampella A, Monti MC, Fiorucci S (2017). Gpbar1 agonism promotes a Pgc-1α-dependent browning of white adipose tissue and energy expenditure and reverses diet-induced steatohepatitis in mice. Sci. Rep..

[61] Carino A, Biagioli M, Marchianò S, Fiorucci C, Zampella A, Monti MCC, Scarpelli P, Ricci P, Distrutti E, Fiorucci S (1864). Ursodeoxycholic acid is a GPBAR1 agonist and resets liver/intestinal FXR signaling in a model of diet-induced dysbiosis and NASH. Biochim. Biophys. Acta Mol. Cell Biol. Lipids.

[62] Harms MH, van Buuren HR, Corpechot C, Thorburn D, Janssen HLA, Lindor KD, Hirschfield GM, Parés A, Floreani A, Mayo MJ, Invernizzi P, Battezzati PM, Nevens F, Ponsioen CY, Mason AL, Kowdley KV, Lammers WJ, Hansen BE, van der Meer AJ (2019). Ursodeoxycholic acid therapy and liver transplant-free survival in patients with primary biliary cholangitis. J. Hepatol..

[63] Marchianò S, Biagioli M, Roselli R, Zampella A, Di Giorgio C, Bordoni M, Bellini R, Urbani G, Morretta E, Monti MC, Distrutti E, Fiorucci S (2022). Beneficial effects of UDCA and norUDCA in a rodent model of steatosis are linked to modulation of GPBAR1/FXR signaling. Biochim. Biophys. Acta Mol. Cell Biol. Lipids.

[64] Ratziu V (2012). Treatment of NASH with ursodeoxycholic acid: Pro. Clin. Res. Hepatol. Gastroenterol..

[65] EminiVeseli B, Perrotta P, De Meyer GRYGRA, Roth L, Van der Donckt C, Martinet W, De Meyer GRY (2017). Animal models of atherosclerosis. Eur. J. Pharmacol..

[66] Fiorucci S, Distrutti E (2021). Linking liver metabolic and vascular disease via bile acid signaling. Trends Mol. Med..

[67] Guillou H, Zadravec D, Martin PGP, Jacobsson A (2010). The key roles of elongases and desaturases in mammalian fatty acid metabolism: Insights from transgenic mice. Prog. Lipid Res..

[68] Straniero S, Laskar A, Savva C, Härdfeldt J, Angelin B, Rudling M (2020). Of mice and men: Murine bile acids explain species differences in the regulation of bile acid and cholesterol metabolism. J. Lipid Res..

[69] Takahashi S, Fukami T, Masuo Y, Brocker CN, Xie C, Krausz KW, Wolf CR, Henderson CJ, Gonzalez FJ (2016). Cyp2c70 is responsible for the species difference in bile acid metabolism between mice and humans. J. Lipid Res..

[70] Chiang JYL (2017). Bile acid metabolism and signaling in liver disease and therapy. Liver Res..

[71] Fiorucci S, Biagioli M, Zampella A, Distrutti E (2018). Bile acids activated receptors regulate innate immunity. Front. Immunol..

[72] Biagioli M, Carino A, Cipriani S, Francisci D, Marchianò S, Scarpelli P, Sorcini D, Zampella A, Fiorucci S (2017). The bile acid receptor GPBAR1 regulates the M1/M2 phenotype of intestinal macrophages and activation of GPBAR1 rescues mice from murine colitis. J. Immunol..

[73] McGavigan AK, Garibay D, Henseler ZM, Chen J, Bettaieb A, Haj FG, Ley RE, Chouinard ML, Cummings BP (2017). TGR5 contributes to glucoregulatory improvements after vertical sleeve gastrectomy in mice. Gut.

[74] Ding L, Sousa KM, Jin L, Dong B, Kim BW, Ramirez R, Xiao Z, Gu Y, Yang Q, Wang J, Yu D, Pigazzi A, Schones D, Yang L, Moore D, Wang Z, Huang W (2016). Vertical sleeve gastrectomy activates GPBAR-1/TGR5 to sustain weight loss, improve fatty liver, and remit insulin resistance in mice. Hepatology.

[75] Ding L, Zhang E, Yang Q, Jin L, Sousa KM, Dong B, Wang Y, Tu J, Ma X, Tian J, Zhang H, Fang Z, Guan A, Zhang Y, Wang Z, Moore DD, Yang L, Huang W (2021). Vertical sleeve gastrectomy confers metabolic improvements by reducing intestinal bile acids and lipid absorption in mice. Proc. Natl. Acad. Sci. U.S.A..

[76] Peiseler M, Schwabe R, Hampe J, Kubes P, Heikenwälder M, Tacke F (2022). Immune mechanisms linking metabolic injury to inflammation and fibrosis in fatty liver disease—Novel insights into cellular communication circuits. J. Hepatol..

[77] Kleiner DE, Brunt EM, Van Natta M, Behling C, Contos MJ, Cummings OW, Ferrell LD, Liu Y-C, Torbenson MS, Unalp-Arida A, Yeh M, McCullough AJ, Sanyal AJ (2005). Design and validation of a histological scoring system for nonalcoholic fatty liver disease. Hepatology.

