# Supplementary material for: Combinatorial therapy with BAR502 and UDCA resets FXR and GPBAR1 signaling and reverses liver histopathology in a model of NASH
Source: Sci Rep. 2023 Jan 28;13:1602. doi: 10.1038/s41598-023-28647-4 (PMC9884292; doi:10.1038/s41598-023-28647-4)
Supplement: Supplementary file 1 — Supplementary Tables. [file 41598_2023_28647_MOESM1_ESM.docx]

Table S1. Genes modulated only by the combination of BAR502 and UDCA (downregulated genes in green, upregulated genes in red).

| ID | HFD-F + UDCA +BAR502 Avg (log2) | HFD-F Avg (log2) | Fold Change HFD-F+UDCA+BAR502 vs HFD-F | P-val |
| --- | --- | --- | --- | --- |
| Ccl3 | 4,87 | 7,19 | -5 | 3,41E-05 |
| Cxcl2 | 2,37 | 4,61 | -4,74 | 1,76E-05 |
| Ccl2 | 3,32 | 5,44 | -4,35 | 7,84E-05 |
| Csf3 | 0,74 | 2,85 | -4,32 | 0,0005 |
| Acod1 | 1,17 | 3,11 | -3,84 | 4,84E-05 |
| Ptgs2 | 0,61 | 2,49 | -3,67 | 0,0006 |
| Ccl7 | 1,39 | 3,19 | -3,48 | 0,0002 |
| Stfa3 | 1,51 | 3,3 | -3,45 | 9,95E-05 |
| Col12a1 | 1,33 | 3,02 | -3,22 | 4,15E-05 |
| Chka | 5,25 | 6,9 | -3,14 | 0,0013 |
| Serpina1e | 9,52 | 11,14 | -3,07 | 0,0093 |
| Npas2 | 1,04 | 2,62 | -2,98 | 0,0008 |
| Itgam | 1,45 | 3,01 | -2,95 | 0,0019 |
| Clec4d | 0,31 | 1,85 | -2,91 | 4,00E-04 |
| Lpl | 3,73 | 5,23 | -2,82 | 9,61E-05 |
| Plin4 | 2,37 | 3,83 | -2,76 | 0,0255 |
| Inhbe | 6,44 | 7,84 | -2,63 | 0,0358 |
| Raet1d | 2,7 | 4,04 | -2,53 | 0,0038 |
| Slc1a4 | 3,03 | 4,28 | -2,38 | 3,92E-05 |
| Pcbp1 | 3,15 | 4,4 | -2,38 | 2,00E-04 |
| Apcs | 6,01 | 7,23 | -2,33 | 0,0038 |
| Rhob | 4,28 | 5,43 | -2,22 | 0,0006 |
| Fxyd2 | 0,74 | 1,89 | -2,22 | 0,0051 |
| G6pc | 10,17 | 11,32 | -2,21 | 0,0347 |
| Ccl4 | 0,91 | 2,04 | -2,2 | 0,0006 |
| Ntrk1 | 0,81 | 1,94 | -2,18 | 0,0012 |
| Slc16a6 | 2,62 | 3,75 | -2,18 | 1,27E-05 |
| Scd1 | 13,52 | 14,64 | -2,18 | 0,0022 |
| Arsg | 4,4 | 5,51 | -2,16 | 0,0022 |
| Nr4a2 | 1,4 | 2,5 | -2,14 | 0,0186 |
| Cyp7b1 | 7,18 | 8,26 | -2,11 | 0,0342 |
| Mup6 | 4,68 | 5,76 | -2,11 | 0,0045 |
| Lgals1 | 6,48 | 7,55 | -2,1 | 0,0056 |
| 1500017E21Rik | 5,22 | 6,28 | -2,09 | 0,0028 |
| Ndufb2 | 4,46 | 5,49 | -2,05 | 0,0029 |
| Cldn1 | 6,34 | 7,37 | -2,04 | 1,07E-06 |
| Clec4e | 0,5 | 1,52 | -2,03 | 0,0072 |
| Mthfr | 3,28 | 4,29 | -2,02 | 0,0009 |
| Sema5b | 3,12 | 4,12 | -2,01 | 0,0002 |

| ID | HFD-F + UDCA +BAR502 Avg (log2) | HFD-F Avg (log2) | Fold Change HFD-F+UDCA+BAR502 vs HFD-F | P-val |
| --- | --- | --- | --- | --- |
| Pdzk1ip1 | 2,19 | 1,18 | 2,01 | 0,0035 |
| Ipmk | 6,36 | 5,35 | 2,01 | 7,80E-06 |
| Spc24 | 6,92 | 5,91 | 2,01 | 3,22E-06 |
| Cbr1 | 7,37 | 6,36 | 2,01 | 0,0153 |
| Hmgcr | 6,22 | 5,2 | 2,02 | 6,39E-05 |
| Plekhg3 | 6,22 | 5,2 | 2,03 | 3,35E-07 |
| Pcsk9 | 6,2 | 5,17 | 2,03 | 1,00E-03 |
| D330041H03Rik | 2,85 | 1,82 | 2,04 | 0,0004 |
| Il6ra | 3,79 | 2,76 | 2,05 | 1,70E-03 |
| Nrg4 | 2,01 | 0,97 | 2,05 | 1,30E-03 |
| Slc46a3 | 4,57 | 3,54 | 2,05 | 0,0005 |
| Gm31649 | 2,3 | 1,26 | 2,06 | 0,0029 |
| Camk2b | 3,93 | 2,89 | 2,06 | 0,0214 |
| Lpin2 | 8,84 | 7,79 | 2,07 | 0,005 |
| Arhgef26 | 5,94 | 4,89 | 2,08 | 5,00E-04 |
| Gm7607 | 4,14 | 3,07 | 2,1 | 0,0045 |
| Plpp6 | 3,85 | 2,77 | 2,11 | 0,0009 |
| Zdhhc3 | 5,69 | 4,61 | 2,11 | 0,0027 |
| Lamc3 | 2,23 | 1,15 | 2,12 | 0,0004 |
| BC023105 | 4,26 | 3,17 | 2,12 | 0,0043 |
| A630034I12Rik | 2,5 | 1,38 | 2,17 | 0,006 |
| Arrdc2 | 4,91 | 3,79 | 2,18 | 0,002 |
| Ttc39aos1 | 3,92 | 2,79 | 2,2 | 0,0388 |
| Meg3 | 4,18 | 3,03 | 2,22 | 1,00E-02 |
| Lrtm1 | 3,2 | 2,05 | 2,22 | 0,0041 |
| Tnfaip8l3 | 2,09 | 0,93 | 2,23 | 0,0016 |
| Upp2 | 8,9 | 7,74 | 2,23 | 0,0026 |
| Sart3 | 4,73 | 3,56 | 2,25 | 0,0002 |
| Bmf | 4,46 | 3,29 | 2,26 | 0,0113 |
| Chmp4c | 2,45 | 1,27 | 2,27 | 0,0001 |
| Map2k6 | 4,92 | 3,73 | 2,28 | 7,49E-07 |
| Acacb | 7,5 | 6,31 | 2,28 | 7,00E-04 |
| Fam13a | 6,24 | 5,04 | 2,29 | 0,0004 |
| Cyp39a1 | 4,39 | 3,17 | 2,32 | 0,046 |
| Tef | 4,94 | 3,71 | 2,35 | 0,0002 |
| Ttc39a | 3,5 | 2,26 | 2,36 | 3,24E-02 |
| Fam57a | 1,92 | 0,67 | 2,38 | 3,40E-05 |
| Tsc22d3 | 4,57 | 3,28 | 2,45 | 0,0149 |
| Ppargc1a | 6,05 | 4,75 | 2,45 | 0,0003 |
| Zfp445 | 6,04 | 4,73 | 2,48 | 0,0002 |
| Sqle | 3,84 | 2,46 | 2,6 | 0,0083 |
| Tmx1 | 5,65 | 4,25 | 2,64 | 1,90E-05 |
| Chrna4 | 5,51 | 3,95 | 2,94 | 1,79E-02 |
| Senp1 | 3,62 | 2,05 | 2,97 | 0,0002 |
| Adgrf1 | 2,17 | 0,57 | 3,04 | 0,0263 |
| 1810008I18Rik | 6,16 | 4,44 | 3,29 | 2,17E-06 |
| Saa4 | 8,86 | 7,02 | 3,57 | 0,0002 |
| Ralbp1 | 3,4 | 1,36 | 4,12 | 1,75E-05 |
| Zbtb16 | 4,25 | 2,19 | 4,17 | 2,30E-03 |
| Rgs16 | 7,91 | 5,38 | 5,78 | 1,53E-02 |

Table S2 Genes modulated by the combination of BAR502 and UDCA and BAR502 alone (downregulated genes in green, upregulated genes in red).

.

| ID | HFD-F + UDCA +BAR502 Avg (log2) | HFD-F + BAR502 Avg (log2) | HFD-F Avg (log2) | Fold Change HFD-F + UDCA +BAR502 vs HFD-F | P-val | Fold Change HFD+BAR502 vs HFD | P-val |
| --- | --- | --- | --- | --- | --- | --- | --- |
| Mogat1 | 1,52 | 2,32 | 3,44 | -3,79 | 3,28E-05 | -2,17 | 0,0044 |
| Cyp2c69 | 2,6 | 2,21 | 4,19 | -3,01 | 4,70E-03 | -3,95 | 6,30E-03 |
| Arntl | 1,66 | 1,89 | 3,19 | -2,9 | 3,00E-04 | -2,46 | 0,0316 |
| Ces4a | 2,93 | 2,6 | 4,4 | -2,76 | 0,015 | -3,48 | 6,10E-03 |
| Rnase2a | 0,52 | 0,4 | 1,95 | -2,7 | 4,49E-02 | -2,93 | 1,66E-02 |
| Rarres1 | 5,82 | 5,77 | 7,09 | -2,41 | 0,0024 | -2,5 | 0,001 |
| Cyp4a10 | 8,06 | 7,4 | 9,19 | -2,19 | 0,0063 | -3,45 | 1,95E-05 |
| Fcor | 1,24 | 1,24 | 2,24 | -2 | 3,41E-05 | -2 | 9,80E-05 |
| Rian | 2,37 | 2,31 | 1,22 | 2,21 | 4,10E-03 | 2,12 | 2,88E-02 |
| Gsta4 | 4,2 | 4,17 | 2,99 | 2,32 | 2,44E-06 | 2,27 | 1,89E-06 |
| Cyp2a5 | 10,31 | 10,31 | 9,05 | 2,4 | 2,80E-05 | 2,4 | 2,51E-05 |
| Cyp2c55 | 3,84 | 3,89 | 2,57 | 2,41 | 0,0002 | 2,49 | 4,62E-05 |
| Obp2a | 4,32 | 4,33 | 2,99 | 2,52 | 0,0289 | 2,55 | 0,0138 |
| Ppl | 5,14 | 5,16 | 3,8 | 2,54 | 0,0005 | 2,58 | 0,0013 |
| Cyp3a59 | 6,53 | 6,77 | 5,19 | 2,55 | 2,30E-03 | 2,99 | 2,00E-04 |
| H2-Q1 | 4,48 | 4,38 | 3,06 | 2,68 | 0,0019 | 2,51 | 3,50E-03 |
| Wee1 | 3,97 | 3,71 | 2,43 | 2,9 | 0,0002 | 2,44 | 6,00E-03 |
| Gstm3 | 4,76 | 4,51 | 3,14 | 3,06 | 0,0096 | 2,57 | 0,0036 |
| Abcb1a | 2,94 | 2,44 | 1,19 | 3,36 | 6,95E-07 | 2,38 | 1,07E-05 |
| Cyp3a44 | 9,43 | 9,75 | 7,66 | 3,4 | 6,83E-05 | 4,26 | 2,19E-06 |
| Cib3 | 5,91 | 5,18 | 3,91 | 4 | 0,0141 | 2,41 | 3,29E-02 |
| Cyp3a11 | 11,44 | 11,37 | 9,39 | 4,14 | 6,27E-06 | 3,96 | 6,72E-07 |
| Gm35287 | 3,2 | 2,51 | 1,05 | 4,44 | 3,84E-09 | 2,75 | 1,67E-06 |

Table S3 Genes modulated by the combination of BAR502 and UDCA and UDCA alone (downregulated genes in green, upregulated genes in red).

| ID | HFD-F + UDCA +BAR502 Avg (log2) | HFD-F + UDCA Avg (log2) | HFD-F Avg (log2) | Fold Change HFDF+UDCA+BAR502 vs HFD-F | P-val | Fold Change HFDF+UDCA vs HFD-F | P-val |
| --- | --- | --- | --- | --- | --- | --- | --- |
| Wrn | 3,21 | 2,72 | 1,35 | 3,64 | 0,0004 | 2,59 | 0,0064 |
| Insig2 | 5,52 | 5,78 | 7,06 | -2,89 | 0,0092 | -2,42 | 0,0106 |
